# Research on the role of gut microbiota metabolites in autism by multi-omics and network pharmacology

**DOI:** 10.3389/fmicb.2026.1811435

**Published:** 2026-08-04

**Authors:** Xiaoyun Hu, Yuning Zeng, Junjie Li, Qiongxi Lin, Zixuan Liang, Jing Zhang, Meirong Jiang, Shuoshuo Gao, Hua Liu

**Affiliations:** 1 Department of Pediatrics, The First Affiliated Hospital of Guangzhou University of Chinese Medicine, Guangzhou, Guangdong, China; 2 The First Clinical Medical College, Guangzhou University of Chinese Medicine, Guangzhou, Guangdong, China; 3 College of Computer Science, Guangdong Polytechnic Normal University, Guangzhou, Guangdong, China; 4 Department of Microbiology, Guangdong Provincial Key Laboratory of Tropical Disease Research, School of Public Health, Southern Medical University, Guangzhou, China; 5 Department of Emergency, The First Affiliated Hospital of Guangzhou University of Chinese Medicine, Guangzhou, Guangdong, China

**Keywords:** 16S rRNA sequencing, autism spectrum disorder, gut-brain communication, metabolomics, network pharmacology

## Abstract

**Objective:**

The relationship and underlying mechanisms linking the gut microbiota, metabolites and autism spectrum disorder (ASD) have not been fully elucidated.

**Methods:**

In the present study, network pharmacology was combined with machine learning for a systematic investigation into the possible action mechanisms between metabolites derived from the gut microbiota and their targets in ASD. Subsequently, untargeted metabolomics analysis and 16S rRNA sequencing were utilized for investigating alterations in differential metabolites and the composition of principal gut microbiota between normal and BTBR mice.

**Results:**

The findings demonstrated that five characteristic targets: CXCR3, HCAR2, HTR1A, IL-6 and NFKB1 modulated by the gut microbiota and gut microbiota’s metabolites were significantly associated with ASD. The M-S-M-T network prioritized 21 candidate gut-microbiota-derived metabolites, including phenylacetic acid and coumarin. These candidates were generated from database-based target prediction and should be interpreted separately from the differential metabolites detected in fecal metabolomics. Metabolomic and 16S rRNA sequencing analyses revealed that BTBR mice displayed metabolic dysregulation and disrupted gut microbiota composition compared with normal ones. These findings suggest that phenylalanine metabolism and neuroactive ligand-receptor interaction may be associated with ASD-like behavioral phenotypes in BTBR mice and warrant further experimental validation. The gut microbiota-metabolite-behavior network analysis further uncovered significant associations among the behavioral characteristics of ASD, alterations in gut microbiota composition and markedly altered metabolite profiles.

**Conclusion:**

By integrating network pharmacology, transcriptomic re-analysis, fecal metabolomics and 16S rRNA sequencing, this study identifies candidate microbe-metabolite-target associations related to ASD-like phenotypes. These results provide testable hypotheses for future mechanistic studies of gut-brain communication in ASD.

## Introduction

As a complicated neurodevelopmental condition, autism spectrum disorder (ASD) features social communication impairment and repetitive and restricted behaviors. Reportedly, over 1% of the global population is affected by ASD (Willsey et al., 2022). The common symptoms of ASD encompass impaired social communication and interaction, excessive repeated behaviors and sensory abnormalities, among others. Moreover, ASD is regarded as a multifactorial disorder caused by the complex interplay of genetic, immune, environmental and metabolic factors. Notably, multiple lines of evidence indicate that the gut microbiota and its metabolites are important in a variety of mental disorders (Bu et al., 2025). Further research is required for a deeper understanding of the associations of the gut microbiota with its metabolites.

Numerous studies have confirmed that the gut-brain axis exerts a great influence on ASD (Wu et al., 2025). This axis highlights the relationships between neurological dysfunction, the dysbiosis of the gut microbiota, alterations in metabolic profiles, and behavioral changes. Signals from the gut microbiota can affect the pathogenesis of neurological disorders via the modulation of inflammation, metabolic pathways and neural signaling (Biagioli et al., 2025). The structure and composition of the gut microbiota were reported to differ greatly from those in the general population. The most pronounced changes were observed in the relative abundances of the major bacterial phyla, *Bacteroidota* and *Firmicutes* (*Bacillota*) (Alharthi et al., 2022). An animal study demonstrated that germ-free mice would show symptoms akin to ASD when the gut microbiota from mice with ASD was transplanted into germ-free mice. The finding further supports that the gut microbiota plays a crucial role in ASD pathogenesis. An animal study of faecal microbiota transplantation indicated that modulating the gut microbiota can regulate the levels of short-chain fatty acids and neurotransmitters in the prefrontal cortex, which ultimately improves ASD symptoms (Zhong et al., 2026). In addition, an imbalance in the gut microbiota is generally accompanied by the dysregulation of its metabolites and associated metabolic pathways. Research indicates that the changes in the abundance levels of *Adlercreutzia* and *Desulfovibrionaceae* are closely correlated with arginine-proline and histidine metabolism (Wang M. et al., 2025). It has also been confirmed that dysbiosis can lead to elevated levels of the aromatic metabolite (p-cresol sulphate) it produces, which further exacerbates neuroinflammation (Zheng et al., 2021). These findings may offer a novel perspective: the gut microbiota and its metabolites could influence ASD initiation and progression, which potentially represent a new intervention target.

Currently, although gut microbiota and their metabolites have been confirmed to be associated with ASD, the following critical mechanistic gaps remain to be filled: first, the association between multi-omics data and behavioral phenotypes remains unclear; second, existing studies lack experimental exploration of the microbe-metabolite-target network. To our knowledge, few studies have jointly integrated network pharmacology, transcriptomic re-analysis, fecal metabolomics, 16S rRNA sequencing, and behavioral phenotyping to investigate candidate gut microbiota-metabolite-target associations in ASD.

**Figure 1:**
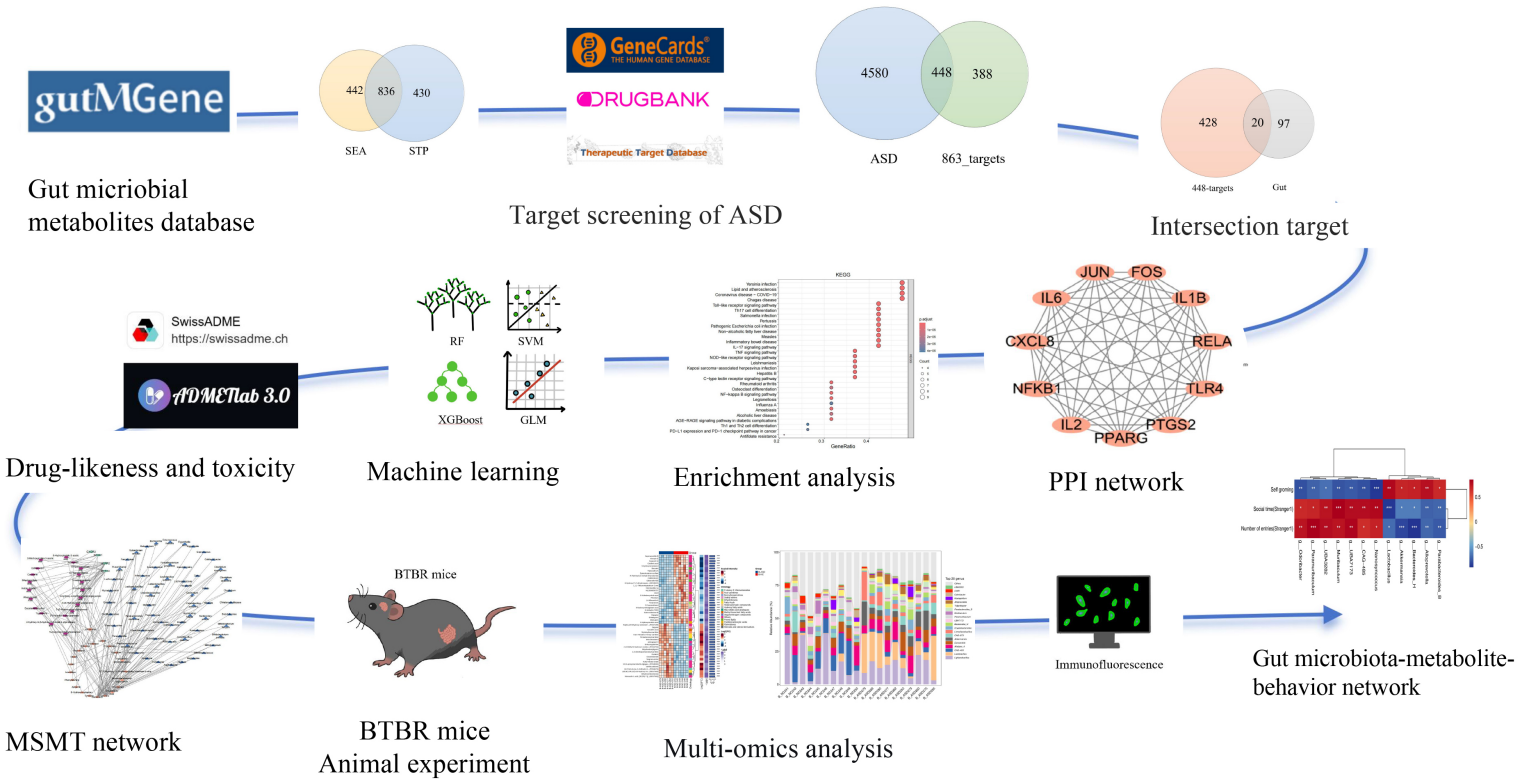
Workflow of study on the role of gut microbiota metabolites in autism by multi-omics and network pharmacology.

In this study, a combination was made of multiple databases, machine learning, untargeted metabolomics and 16S ribosome ribonucleic acid (rRNA) gut microbiota sequencing. Additionally, gut microbiota-substrate-metabolite-target (M-S-M-T) and gut microbiota-metabolite-behavior networks were constructed. This approach revealed the pathological mechanism underlying ASD initiation and progression. The findings of this study indicate that phenylacetic acid and coumarin are key gut microbiota metabolites. Five core targets (chemokine (C-X-C motif) receptor 3 (CXCR3), hydroxycarboxylic acid receptor 2 (HCAR2), 5-hydroxytryptamine receptor 1A (HTR1A), interleukin-6 (IL-6) and nuclear factor kappa B subunit 1 (NFKB1)) regulated by the gut microbiota and its metabolites are possibly novel targets for the treatment of ASD. Furthermore, through untargeted metabolomics, animal experiments and 16S rRNA gut microbiota sequencing, strong correlations were revealed among the behavioral performance of ASD, alterations in the composition of the gut microbiota, and significant differential metabolites. Several gut bacterial genera, including *g_Alloprevotella*, *g_Bacteroides_H* and *g_Parabacteroides_B*, were associated with ASD-like behavioral and metabolic features in the BTBR mouse model. It may also be attributable to phenylalanine metabolism, neuroactive ligand-receptor interaction and other key metabolic pathways. Collectively, these initial results not only shed light on the therapeutic value of gut microbiota metabolites but also provide important clues regarding potential gut-brain communication in ASD. Figure 1 presents the overall workflow.

## Methods

### Data sources

The autism spectrum disorder (ASD) transcriptomic dataset GSE18123 was downloaded from the Gene Expression Omnibus (GEO) database (Barrett et al., 2013).^1^ The complete GSE18123 series comprises 285 peripheral blood samples (170 ASD cases and 115 healthy controls) distributed across two microarray platforms. In the present study, the GPL6244 subset, consisting of 104 ASD cases and 82 healthy controls, was selected for downstream analyses because of its larger sample size and gene-level expression profiles, which improve the statistical power and reliability of subsequent bioinformatics analyses.

Targets of gut microbiota metabolites:

Metabolites derived from the human gut microbiota were retrieved from the gutMGene database (Qi et al., 2025).^2^ After the removal of duplicates, these metabolites were submitted to the PubChem database (Kim et al., 2023).^3^ Thus, their corresponding simplified molecular input line entry system (SMILES) notations were acquired. Then, target prediction was achieved by importing the SMILES strings into Swiss Target Prediction (Shang et al., 2023) (STP)^4^ and Similarity Ensemble Approach (Wang et al., 2016) (SEA)^5^ databases. The species parameter was set as “*Homo sapiens*.”

Gut targets:

Targets related to the human gut were acquired from the gutMGene database.

Disease targets of ASD:

“ASD” was taken as the key term to search potential disease-associated targets in GeneCards (Safran et al., 2010),^6^ DrugBank (Knox et al., 2024),^7^ and Therapeutic Target Database (Zhou et al., 2024) (TTD)^8^ databases. In GeneCards, only genes encoding proteins with a relevance score of > 5 were kept. A unified set of ASD-related targets designated as ASD_targets was formed by merging and deduplicating the targets identified from the above-mentioned three databases.

### Identification of predicted targets

Potential gut microbiota metabolite targets were retrieved from SEA and STP databases. After duplicates were removed, the intersection of these targets was designated as the targets related to gut microbiota metabolites. Subsequently, these targets were intersected with disease-associated targets for the identification of disease-relevant gut microbiota metabolite targets. Finally, the resulting targets were further intersected with targets derived from the gut to obtain an ultimate set of predicted targets for subsequent analyses. Cytoscape software (Shannon et al., 2003) (version 3.9.1) was used to construct a disease–intersecting target–gut microbiota tripartite network.

### Protein–protein interaction network construction and analysis

The protein–protein interaction (PPI) network was constructed by uploading the key targets of ASD and the intersecting proteins of gut microbiota metabolites to the STRING database (Szklarczyk et al., 2023).

### Gene ontology and Kyoto encyclopedia of genes and genomes enrichment analyses

The DAVID database was used to subject the intersecting proteins to Gene Ontology (GO) and Kyoto Encyclopedia of Genes and Genomes (KEGG) enrichment analyses. The visualization of the results was performed through a bioinformatics platform. GO terms and KEGG pathways that had a *p*-value of less than 0.05 were initially selected to conduct statistical analysis. Afterwards, it was considered that terms with a false discovery rate (FDR) of less than 0.05 showed statistical significance.

### Predictive model construction on the basis of multiple machine learning methods

The GSE18123_GPL6244 dataset was utilized for evaluating the expression levels of the previously identified predicted targets between the ASD and control groups. Differential expression analysis was carried out using the Wilcoxon rank-sum test with a significance threshold of *p* < 0.05. Genes that demonstrated statistically significant differences were chosen as explanatory variables for subsequent machine learning modeling.

R packages caret (v6.0–94) and xgboost (v1.7.7.1) were leveraged to construct four machine learning models, namely Random Forest (RF), Generalized Linear Model (GLM), Support Vector Machine (SVM) and eXtreme Gradient Boosting (XGBoost). Machine-learning models were developed and compared within the GSE18123_GPL6244 dataset using receiver operating characteristic (ROC) curve analysis and the corresponding area under the curve (AUC). To explain and compare model performance, the DALEX package (v2.4.3) was employed for explainable artificial intelligence (XAI) visualizations, including residual boxplots, reverse cumulative distribution of residuals and receiver operating characteristic (ROC) curves.

The optimal model was selected based on the comprehensive evaluation of discrimination ability, calibration and stability. At last, the five most critical variables determined by feature contribution metrics were extracted from the best-performing model as characteristic biomarkers for further investigation.

### Expression and ROC validation of characteristic targets

The expression levels of characteristic targets were visualized based on the GSE18123_GPL6244 dataset. The pROC package (Robin et al., 2011) (version 1.18.5)^9^ was adopted to plot ROC curves and calculate the area under the curve (AUC) to assess the predictive performance of each gene. Moreover, Pearson’s correlation algorithm was applied to evaluate pairwise correlations among characteristic targets. The results were expressed as a correlation heatmap.

### Association between immune infiltration and characteristic genes

For the purpose of characterizing the immune microenvironment in ASD, immune infiltration profiles were evaluated between ASD and control groups using the GSE18123_GPL6244 dataset. Implemented in the gsva function of the GSVA package (Hänzelmann et al., 2013) (version 2.2.0),^10^ the single-sample GSE analysis (ssGSEA) algorithm was utilized for quantifying the activity of immune cells in samples. The Wilcoxon rank-sum test was used to identify immune cell types demonstrating significant between-group differences (*p* < 0.05).

Pairwise Spearman correlation analyses were conducted for evaluating associations not only among immune cells but also between immune cells and characteristic genes utilizing the psych package (version 2.4.1).^11^ The ggplot2 package (version 3.5.2)^12^ was used to visualize resulting correlation patterns in the form of heatmaps and lollipop plots.

### Single-gene GSEA

GSEA was performed for single genes on the basis of KEGG and GO gene sets in the software GSEA (version 3.0), with a view to exploring the underlying function of biomarkers. GO enrichment analysis primarily depicted the biological processes (BPs), cellular components (CCs) and molecular functions (MFs) correlated with genes. The correlation coefficients of each gene with all genes in gene sets were ranked according to the expression value of each gene. GSEA significance was assessed using both the nominal (NOM) *p* value and the false discovery rate (FDR) q-value. Pathways with NOM *p* < 0.05 and FDR q < 0.25 were considered significantly enriched.

### Evaluation of drug-likeness and toxicity

SwissADME^13^ (Daina et al., 2017) and ADMETlab^14^ (Fu et al., 2024) platforms were used for assessing the drug-likeness and toxicity of the identified key metabolites, respectively. Lipinski’s Rule of Five can be applied to preliminarily evaluate molecules in the early phases of drug design and screen candidate high-potential drug molecules. This approach helps save time and resources, which thus makes the development of drugs more efficient. The following Lipinski’s Rule of Five is used to determine drug-likeness: (1) Molecular weight is below 500; (2) Lipid-water partition coefficient is below 5; (3) The count of hydrogen bond acceptors is below 5; (4) The count of hydrogen bond donors is below 5; (5) Polar surface area is below 140. Metabolites satisfying the criteria of Lipinski’s Rule of Five were chosen for further analysis.

### Analysis of the “microbiota-substrate-metabolite-target” network

A “microbiota-substrate-metabolite-target” (M-S-M-T) network was constructed for elucidating their complex relationship. Cytoscape 3.9.1 was employed for the visualization of the M-S-M-T network.

### Animals

Ten male C57BL/6 J mice and ten male BTBR T + tf/J (BTBR) male mice aged 4–6 weeks were provided by Jiangsu Aniphe Biolaboratory Inc. Co., Ltd. (Animal Production License Number: SCXK(SU)2023-0020, Jiangsu). All mice were raised under specific pathogen-free conditions with 50 ± 10% humidity and a 12-h light/dark cycle at a temperature of 22–24 °C. They were allowed to had ad libitum access to food and water. All animal experiments followed the National Institute of Health Guide for the Care and Use of Laboratory Animals. Animal experiment procedures were permitted by the Experimental Animal Ethics Committee of Guangzhou University of Chinese Medicine (No. 20260309061). Referencing prior literature (Liu et al., 2024), mice underwent behavioral testing at 6–10 weeks of age in the following order: open field exploration, social interaction, and self-grooming behavior, with at least 5-day intervals between each test. Both tissue and fecal samples were collected after all behavioral tests were completed.

A total of 10 C57BL/6 J mice and 10 BTBR mice were included in this study. All animals were used for 16S rRNA sequencing analysis. According to the experimental design, behavioral assessments and fecal metabolomics analyses were performed using six mice per group, whereas immunofluorescence (IF) staining and RT-qPCR validation were conducted using tissue samples from three mice per group. Therefore, the sample sizes differed among experimental procedures because different subsets of animals were allocated to specific analyses according to the study design, rather than as a result of animal attrition or *post hoc* exclusion after data collection.

### Three-chamber social interaction test

The social approach was tested in a three-chambered apparatus. After 10-min habituation, a mouse of the same strain, sex and age (Stranger 1) was placed in one side of the chamber, and a new object was placed on the other side. After both stimuli were positioned, subject mice were placed in the central compartment. Social behavior was recorded by trained observers over 10 min: the number of entries into different compartments and the time spent in every compartment.

### Self-grooming behavior test

All mice were arranged in a clean empty cage for 10 min of habitation. After the commencement of the formal experiment, the self-grooming behaviors of mice were recorded for 10 min, including head washing, body cleaning, genital/tail cleaning, and paw and leg licking.

### Open field test

A rectangular box with dimensions of 50 cm × 50 cm × 40 cm was used for the open field test. During the adaptation phase, experimental mice were placed in the center of the arena for 10-min free exploration. In the experimental phase, the traveling distance and dwell time of mice were observed and recorded in 5 minutes.

### Metabolomics analysis of fecal samples

A UPLC-ESI-Q-Orbitrap-MS system (Shimadzu Nexera X2 LC-30 AD, Shimadzu, Japan), together with Q-Exactive Plus (Thermo Scientific, San Jose, USA), was used to analyze the metabolomics profiling of fecal samples of all groups. Models of metabolic profiles were grounded in orthogonal partial least-square discriminant analysis (PLS-DA), principal component analysis (PCA) and partial least-square discriminant analysis (OPLS-DA). All the evaluated models were tested for over-fitting with permutation test methods. A statistically significant threshold of variable influence on projection (VIP) values acquired from the OPLS-DA model was used to get discriminating metabolites. One-way analysis of variance (ANOVA) was utilized to calculate the *p* value for multiple group analysis. It was considered that metabolites with VIP values above 1.0 and p value below 0.05 showed statistical significance.

### Gut microbiota analysis

Total deoxyribonucleic acid (DNA) was extracted from the faecal samples of each mouse group. Specific primers were used to amplify various regions of the 16S rRNA gene in bacteria via polymerase chain reaction (PCR). Magnetic beads (Vazyme VAHTSTM DNA Clean Beads) were utilized for the purification of PCR products. TruSeq Nano DNA LT Library Prep Kit (Illumina) was employed to construct sequencing libraries. Then, the NovaSeq sequenceron was used for the high-throughput sequencing of the gut microbiota. After sequencing, raw data were analyzed and operational taxonomic units (OTUs) were clustered by Vsearch. Subsequent bioinformatics analysis involved assessing alpha (*α*) diversity, beta (*β*) diversity and differential species abundance.

### Immunofluorescence analysis

Following perfusion, brain tissues comprising the cortex and hippocampus were carefully dissected and prepared for histological examination. The prefrontal cortex and hippocampus tissue sections were fixed with 4% paraformaldehyde. Then, tissues were paraffin-embedded and sectioned, followed by the deparaffinization, rehydration and antigen retrieval of tissue sections. To reduce nonspecific binding, a 5% solution of bovine serum albumin (BSA) in phosphate buffer saline (PBS) was used to block sections. Next, sections were incubated with primary antibodies CXCR3 (1: 200, Immunoway, #YT5277), HCAR2 (1: 100, Affinity, #DF4890), HTR1A (1: 200, Immunoway, #YT4391), IL-6 (1: 100, Servicebio, #GB11117) and NFKB1 (1: 100, Immunoway, #YM8298) at 4 °C overnight. Afterwards, samples were treated with fluorescent secondary antibodies (1:400, SeraCare, #5220–0336). To visualize the cell nucleus, dihydrochloride (DAPI) was added for staining. After staining, a laser scanning confocal microscope was leveraged to capture images.

### RT-qPCR analysis

Total RNA was extracted from the hippocampus and cortex using TRIzol reagent. RT-qPCR was performed on the Quantagene q225 Real-Time Quantitative PCR System using Evo M-MLV RT Mix Kit with gDNA Clean for qPCR and SYBR Green Master Mix. The specific primers for each gene are listed in Supplementary Table 1. GAPDH was used as the internal reference gene for result calculation.

### Statistical analysis

Each experiment was conducted at least in triplicate. In addition, all data were shown as means ± standard deviations (SDs) and quantitatively analyzed utilizing SPSS (version 26.0) and GraphPad Prism (Version 9). The independent samples *t*-test, Welch’s *t*-test and two-way mixed-design repeated-measures ANOVA were utilized to conduct parametric analyses as appropriate. For quantitative analysis of social behavior, a two-way mixed-design repeated-measures ANOVA was performed, with group (B6J or BTBR) as the between-subjects factor and cage type (Stranger 1 or Empty) as the within-subjects factor. When the main effects or interaction effects were significant, simple effects analysis was performed subsequently, and LSD test was used for pairwise comparisons within and between groups. A value of *p* ≤ 0.05 was considered statistically significant.

## Results

### Targets of gut microbiota metabolites intervening in ASD have been identified

**Figure 2:**
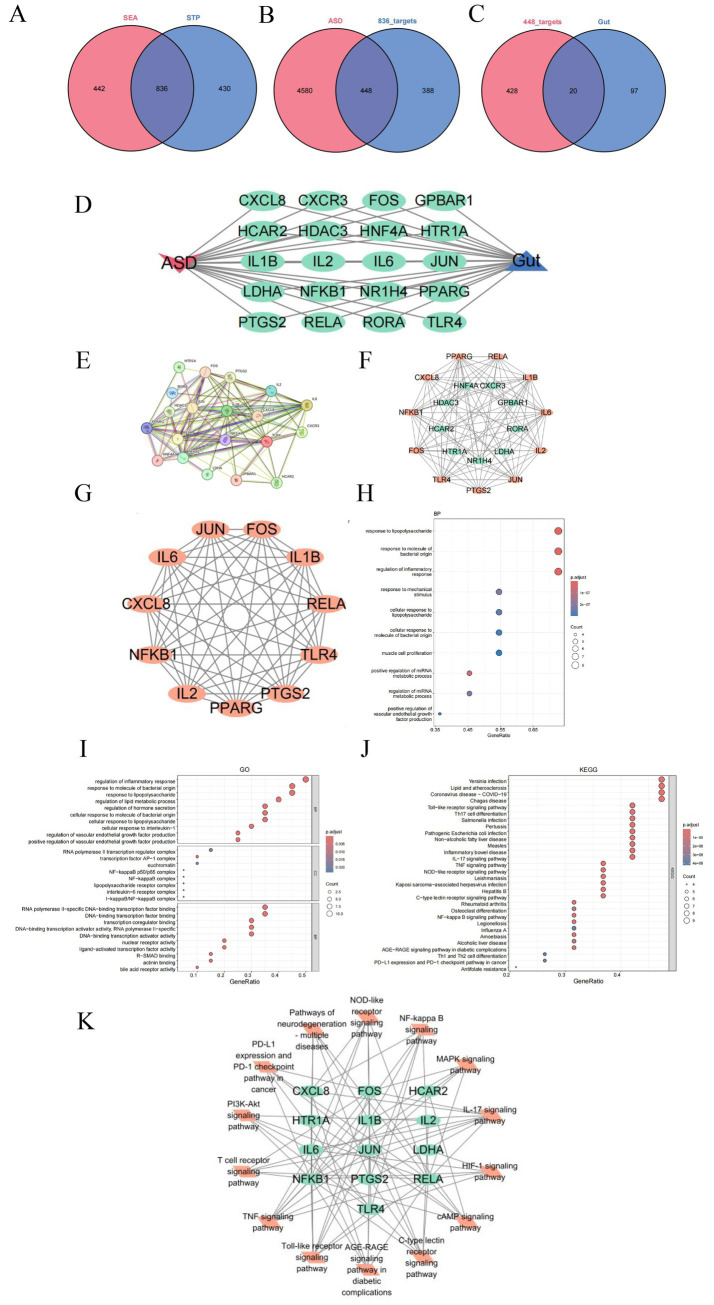
Identification and functional enrichment analysis of genes regulated by gut microbiota metabolites in ASD. **(A)** Venn diagram showing common targets of gut microbiota metabolites by SEA and STP. **(B)** Venn diagram showing overlapping targets between gut microbiota metabolite targets and ASD. **(C)** Venn diagram showing the final common targets between human gut targets and gut microbiota metabolites-ASD. **(D)** Construction and visualization of the gut-target-ASD candidate association network. **(E,F)** Visualization of the protein–protein interaction (PPI) network constructed from the overlapping targets. **(G)** The key functional cluster (Cluster 1) extracted from the PPI network. **(H)** GO biological process enrichment analysis of genes in Cluster 1. **(I,J)** GO and KEGG pathway enrichment analyses of overlapping target genes. **(K)** Visualization of the target–pathway regulatory network. SEA, similarity ensemble approach; STP, Swiss Target Prediction; ASD, autism spectrum disorder.

Firstly, 251 gut microbiota metabolites and 117 human gut targets were obtained from the gutMGene database. Then, 1,266 and 1,278 targets associated with 251 gut microbiota metabolites in SEA and STP databases were identified. The 836 overlapping targets between SEA and STP were deemed as the main targets of 251 gut micro-biota metabolites (Figure 2A). In total, 5,028 targets related to ASD were retrieved from GeneCards, Drugbank and TTD databases. After the 836 common targets of gut microbiota metabolites were intersected with these targets related to ASD, 448 common targets were acquired (Figure 2B). Finally, 20 targets were obtained from the VENN diagram between 448 gut and common targets (Figure 2C). The gut-target-ASD network is presented in Figure 2D. These results revealed that 20 targets take charge of regulating ASD via the gut microbiota.

### Hub genes and functional clusters identified by PPI network analysis

To identify core regulatory targets and functional modules, a protein–protein interaction (PPI) network was constructed based on the 20 overlapping targets (Figures 2E,F). The network topology and modular structure were analyzed using the STRING database and visualized with Cytoscape 3.9.1. Cluster analysis was further performed using the MCODE plugin to explore functionally important gene modules. A prominent functional Cluster 1 containing 11 genes was identified as the core module within the PPI network (Figure 2G). The GO-BP enrichment analysis revealed that Cluster 1 was significantly enriched in 1,160 signaling pathways (adjusted *p*-value < 0.05), including “response to molecules of bacterial origin,” “response to lipopolysaccharides (LPSs),” “regulation of inflammatory responses,” and “positive regulation of micro RNA (miRNA) metabolic process” (Figure 2H). These results indicate that the core targets are strongly associated with inflammatory regulation and microbial signal–related biological processes.

### Enrichment analysis revealed the biological functions and pathways of the targets

GO analysis is a key tool that helps describe the roles of proteins and genes within cells. As a potent tool, KEGG analysis is capable of describing well-characterized biological pathways and predicting the functions of currently unknown proteins or genes. This approach provides valuable insights into the regulatory mechanisms governing BPs, which thus contributes to their overall understanding. Therefore, a significance threshold of adjusted p-value below 0.05 was used to perform GO and KEGG pathway analyses on the 20 overlapping targets.

In total, 1,057 pathways were found to be enriched in the GO_BP. Notable enrichment was observed in pathways including “response to LPSs, “the regulation of inflammatory responses” and “response to molecules of bacterial origin.” In the GO_CC, eight pathways were enriched, with significant representation in the “transcription factor activator protein-1 (AP-1) complex,” “NF-κB p50/p65 complex” and “euchromatin.” GO_MF analysis identified enrichment in 135 pathways, which prominently featured “transcription coregulator binding,” “nuclear receptor activity” and “RNA polymerase II-specific DNA-binding transcription factor binding” (Figure 2I). Additionally, KEGG pathway enrichment analysis revealed 144 pathways, with significant enrichment in “C-type lectin receptor signaling pathway,” “the Toll-like receptor (TLR) signaling pathway” and “the IL-17 signaling pathway” (Figure 2J). After these analyses, a network illustrating the relationships of the identified targets with pathways was constructed and subsequently visualized (Figure 2K).

### RF model prioritized a five-gene signature with moderate internal discrimination

**Figure 3:**
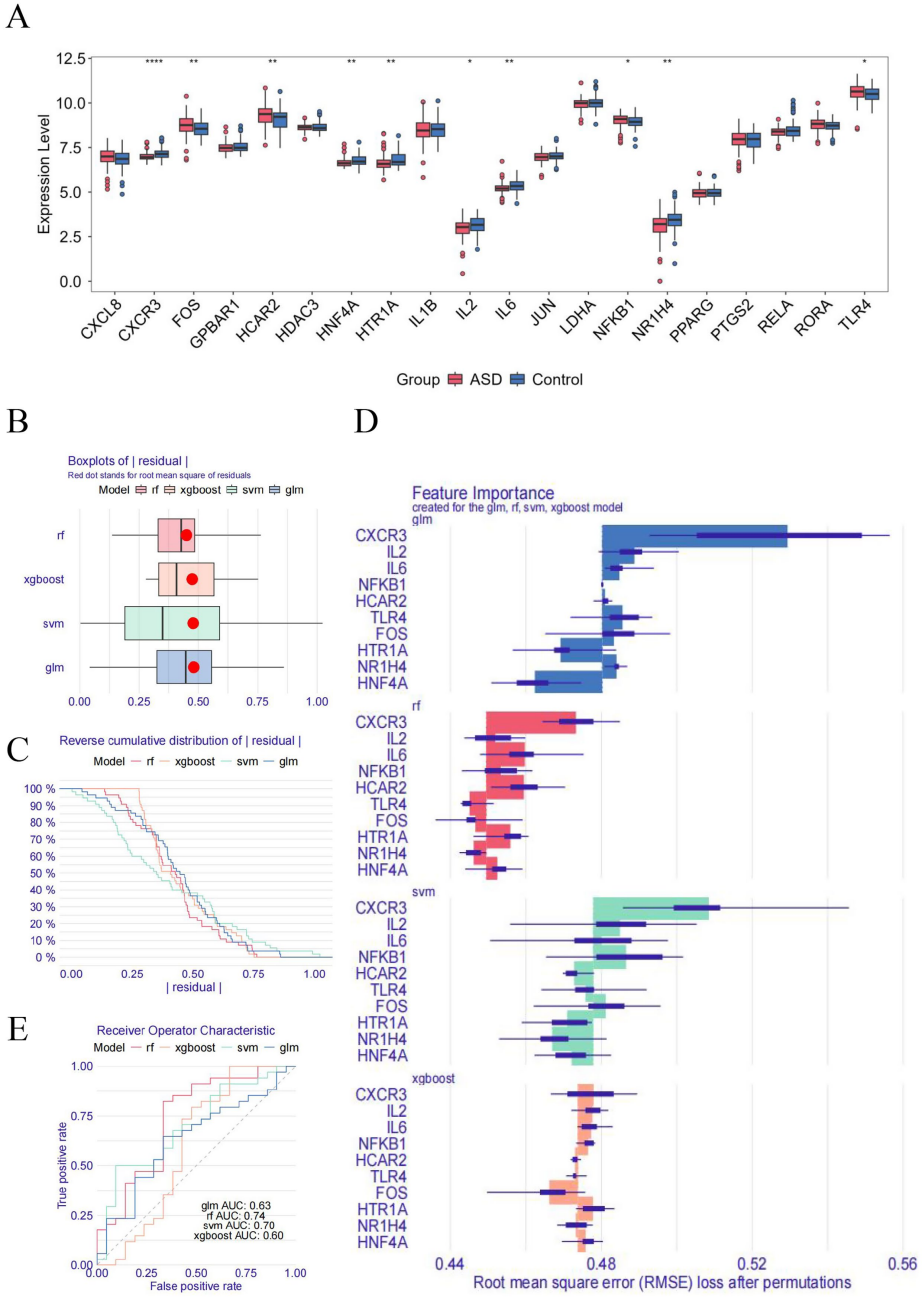
Screening and validation of characteristic genes using machine learning algorithms **(A)** Boxplot showing expression levels of the intersection target genes between ASD and control groups **(B,C)** Residual distribution of four machine learning models. The random forest (RF) model exhibited the lowest residual distribution, indicating superior predictive performance **(D)** Key feature importance ranking in machine learning models. The top 10 genes from each model were ranked according to root mean square error (RMSE) **(E)** ROC curve analysis of the four machine learning models using five-fold cross-validation in the testing cohort. The area under the curve (AUC) values were 0.74 for RF, 0.70 for SVM, 0.60 for XGB, and 0.63 for GLM, respectively.

Four machine learning models: RF, GLM, SVM and XGB, were established for the further identification of specific genes with great diagnostic value. On the basis of GSE18123, the expression differences of the above 20 intersections between the ASD and control groups were analyzed using the Wilcoxon test. The aforesaid four models were explained using the R package “DALEX.” The residual distribution of all models was drawn in the validation set. The results showed that the 10 genes exhibited significant differences between the ASD and control groups, with *p*-values less than 0.05 (Figure 3A). These 10 genes contained TLR4, IL2, IL6, CXCR3, NFKB1, nuclear receptor subfamily 1, group H, member 4 (NR1H4), hepatocyte nuclear factor 4α (HNF4A), FBJ murine osteosarcoma viral oncogene homolog (FOS), HCAR2 and HTR1A.

The RF machine learning model had lower residuals (Figures 3B,C). Subsequently, genes were ranked by feature importance, and the top-ranked genes were selected for subsequent analysis on the top 10 features of every model in accordance with root mean square error (RMSE) (Figure 3D). Additionally, ROC curves were computed to evaluate the discriminative performance of the above-mentioned four machine learning algorithms in light of five-fold cross-validation in the training set (GSE18123 dataset) (Figure 3E). The AUCs of the four models were obtained (RF, SVM, XGB and GLM had an AUC of 0.74, 0.70, 0.60 and 0.63, respectively). Based on the residual error and area under the receiver operating characteristic curve (AUC), the random forest (RF) model showed the best performance in distinguishing different ASD clusters. Therefore, the top five genes ranked by variable importance (CXCR3, IL6, HCAR2, HTR1A, and NFKB1) were selected as candidate feature genes for subsequent analyses.

### The predictive model with five characteristic genes showed favorable performance and CXCR3 as the optimal biomarker

According to the described method, five characteristic gene expression box plots were drawn. The results illustrated in Supplementary Figure 1A indicated that in the GSE18123 dataset, CXCR3, IL6 and HTR1A were expressed at low levels in disease samples, while HCAR2 and NFKB1 were expressed at high levels in disease samples.

The Pearson algorithm was used for calculating the correlations between characteristic genes. A significant correlation was detected between each pair of characteristic genes (Supplementary Figure 1B). HTR1A was observed to have the strongest positive relationship with CXCR3 and IL6, with a correlation value of 0.51. HCAR2 was demonstrated to have the strongest negative association with CXCR3, with a correlation value of −0.52. The ROC curve demonstrated that the predictive model with five core markers showed favorable performance, which indicated the ability of the diagnostic model to effectively discriminate patients with ASD from normal cases. Beyond that, the diagnostic function of single markers was also validated. The AUC of CXCR3 was highest (AUC = 0.7, Supplementary Figure 1C).

### ASD characteristic genes exhibited distinct immune cell infiltration patterns

**Figure 4:**
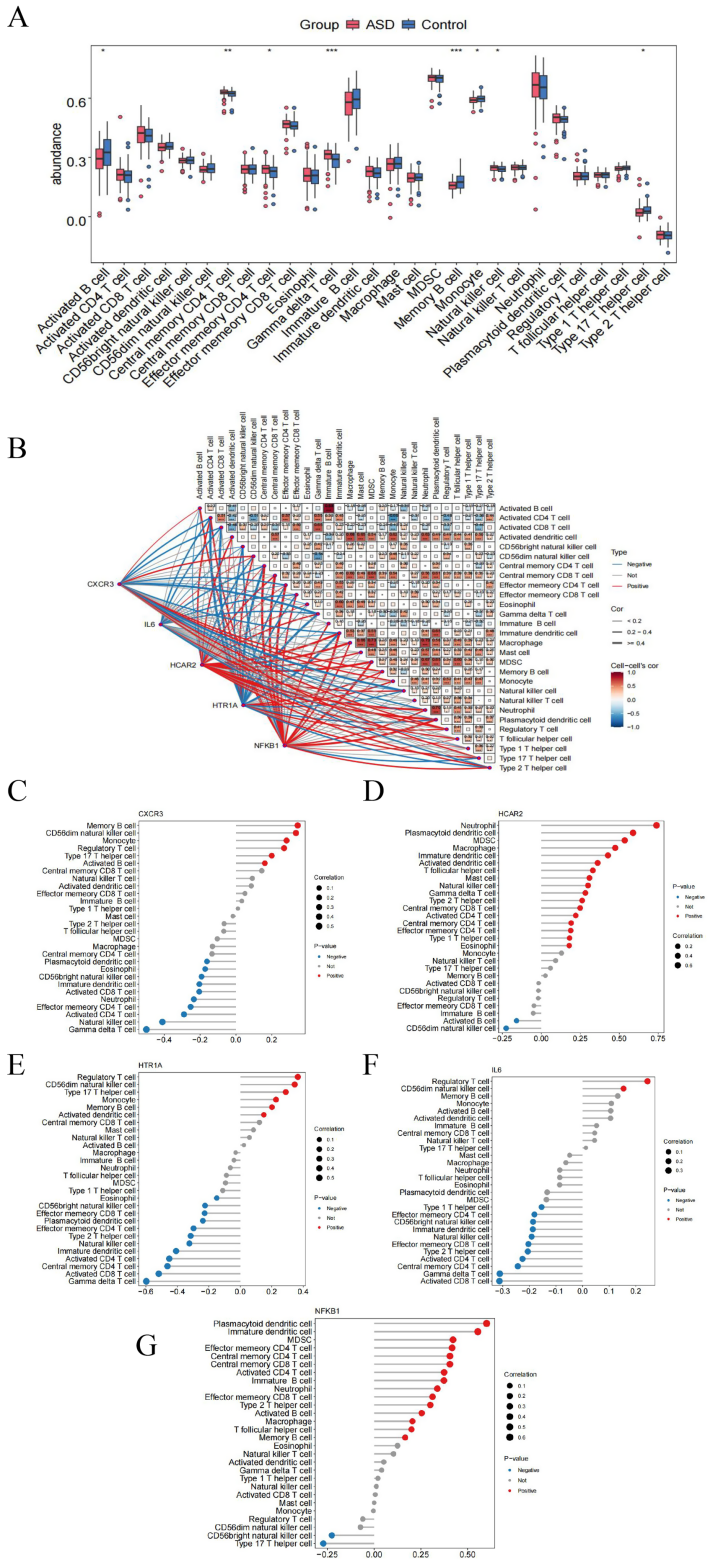
Immune cell infiltration profiles and their correlations with characteristic genes in ASD. **(A)** Comparison of immune cell infiltration proportions between ASD patients and healthy controls using boxplot analysis. **(B)** Correlation matrix showing the interrelationships among 16 types of immune cells and the five characteristic genes. **(C–G)** Visualization of correlations between the five characteristic genes (CXCR3, HCAR2, HTR1A, IL-6, and NFKB1) and individual immune cell subsets.

Here, ssGSEA was used to assess the enrichment scores of different immune cell subpopulations and functions, with the objective of further examining immune cell infiltration and functions between patients with ASD and healthy controls. The visualization of the results was completed via box plots (Figure 4A). ASD patients showed significant differences in activated B cells, memory B cells, monocytes, natural killer cells, gamma delta T cells, central memory cluster of differentiation 4 (CD4) T cells, effector memory CD4 T cells, and type 17T helper (Th) cells. The correlations of eight immune cells showed a positive association between activated immature B cells and activated B cells (*r* = 0.88), and a negative association between monocytes and activated CD4 T cells (*r* = −0.60) (Figure 4B). Among the five characteristic genes, CXCR3, IL6, HCAR2, HTR1A and NFKB1 were strongly linked to immature dendritic cells, activated CD4 T cells and effector memory CD4 T cells, which indicated close associations with various immune cell infiltrations (Figures 4C–G).

### Characteristic genes revealed significant enrichment in key pathways and gene-specific functional involvements

For the purpose of further explaining the biological functions of characteristic genes, characteristic genes were described and analyzed using GSEA for single genes. The enrichment analysis results suggested the significant enrichment of the five characteristic genes in pathways including ‘apoptosis’, ‘neuroactive ligand receptor interaction’, ‘nucleotide excision repair’, ‘the B cell receptor signaling pathway’ and ‘extracellular matrix (ECM)-receptor interaction’ (Supplementary Figure 2).

It was observed that NFKB1, HCAR2, HTR1A, and IL-6 were also implicated in the regulation of ‘olfactory transduction’. Specifically, NFKB1, HCAR2, and HTR1A were involved in the ‘lysosome’ and ‘spliceosome pathways’. Both NFKB1 and CXCR3 showed enrichment in ‘ubiquitin-mediated proteolysis’. Additionally, HCAR2 and CXCR3 were associated with the ‘nucleotide-binding oligomerization domain (NOD)-like receptor signaling pathway’ as well as the ‘TLR signaling pathways’. HTR1A and CXCR3 contributed to the regulation of ‘oxidative phosphorylation’. Notably, CXCR3 was also involved in the regulation of ‘tyrosine metabolism’, ‘linoleic acid metabolism’, ‘focal adhesion’, and ‘cytokine-cytokine receptor interactions’, etc.

### Key metabolites meet Lipinski’s rule of five

The drug-likeness and toxicity of key metabolites were further assessed to make sure that they are safe for therapeutic use. As demonstrated in Supplementary Table 2, main metabolites such as 5-(3-hydroxyphenyl) pentanoic acid, 3-Phenylpropionic acid, 3-Indolepropionic acid, Indole-3-lactic acid, 4-Hydroxybenzoic acid, 5-Methoxyindole-3-acetic acid, 3-(1H-indol-3-yl) propanoate and 3-(3-Hydroxyphenyl) propanoic acid all observe Lipinski’s Rule of Five (Supplementary Table 2). Toxicity tests suggested (Supplementary Table 3) that 3-Phenylpropionic acid, Indole-3-lactic acid, 5-Methoxyindole-3-acetic acid and other key metabolites mentioned in Supplementary Table 3 do not cause carcinogenicity or damage the liver. These results revealed that metabolites such as 3-Indolepropionic acid, Indole-3-lactic acid and 5-Methoxyindole-3-acetic acid contribute positively to gut health.

### The M-S-M-T network revealed close connections among gut microbiota, metabolites, and characteristic target genes in ASD

**Figure 5:**
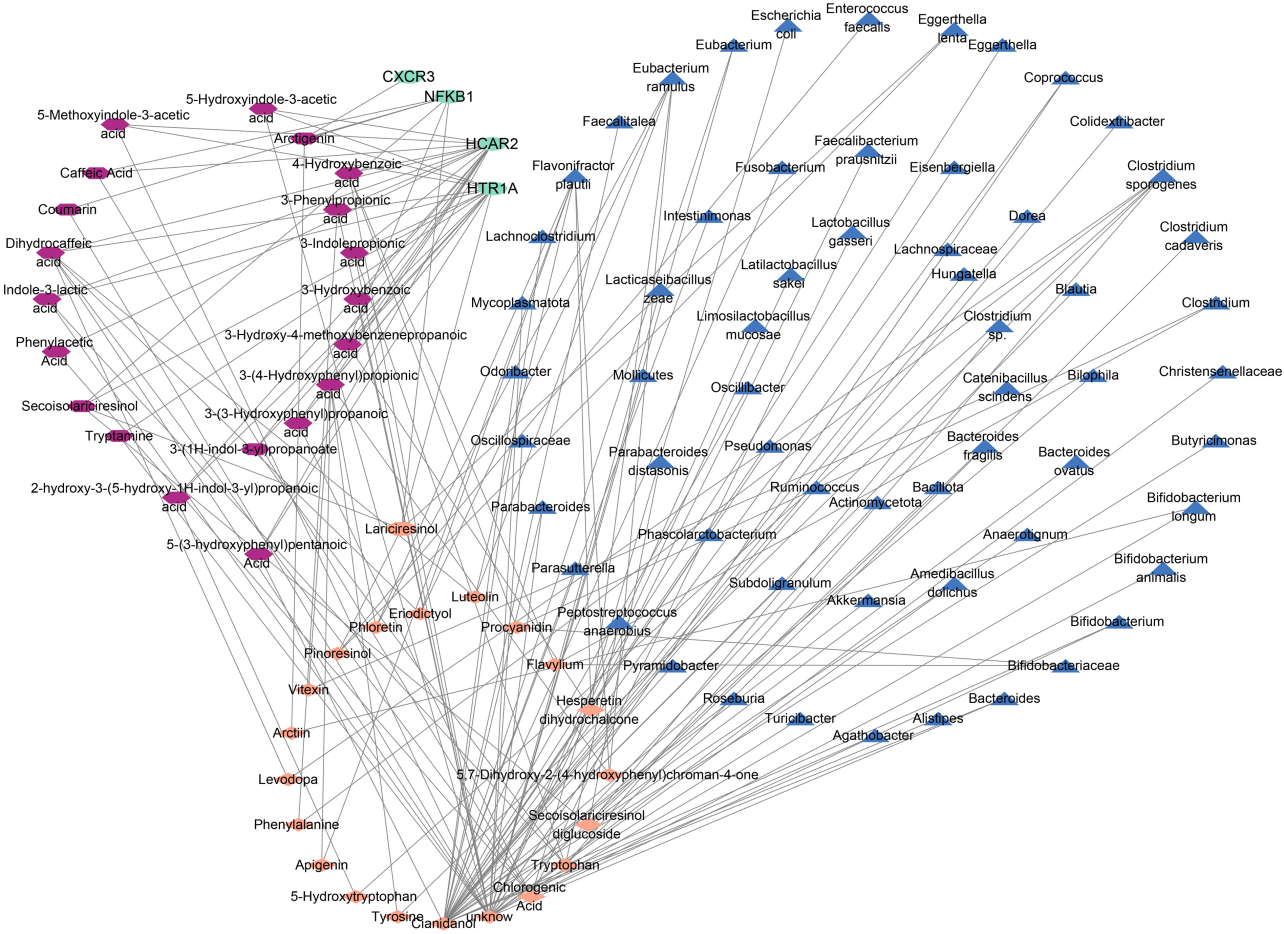
Construction and structure of the microbiota-substrate-metabolite-target (M-S-M-T) regulatory network in ASD.

The construction of an M-S-M-T network illustrated the regulatory framework linking 61 gut microbiota, 21 substrates, 21 non-toxic metabolites and characteristic target genes CXCR3, HCAR2, HTR1A and NFKB1 (Figure 5). This network underscores that these genes, as key mediators, play a pivotal role in microbiota–host interactions in ASD. NFKB1 is associated with 6 metabolites (caffeic Acid, arctigenin, secoisolariciresinol, lariciresinol, 3-Hydroxy-4-methoxybenzenepropanoic acid and coumarin), 10 substrates (chlorogenic Acid, hesperetin dihydrochalcone, arctiin, secoisolariciresinol diglucoside, lariciresinol, pinoresinol, and 4 unidentified), and 15 gut microbial species (e.g., *Bifidobacterium*, *Lactobacillus gasseri*, *Parabacteroides*, and *Enterococcus faecalis*). HTR1A is linked to 15 metabolites, including dihydrocaffeic acid, 3-(3-Hydroxyphenyl)propanoic acid, 3-Indolepropionic acid, Indole-3-lactic acid, Tryptamine, et al., along with 18 substrates (tryptophan, apigenin, pinoresinol, 5-Hydroxytryptophan, chlorogenic acid, et al.) and 54 gut microbiota (*Bacteroides fragilis*, *Parabacteroides*, et al.). HCAR2 interacts with 16 metabolites (2-hydroxy-3-(5-hydroxy-1H-indol-3-yl)propanoic acid, dihydrocaffeic acid, phenylacetic acid, et al.), 17 substrates (phenylalanine, cianidanol, luteolin, et al.), and 55 gut microbiota (*Bifidobacteriaceae*, *Lachnoclostridium*, et al.). Similarly, CXCR3 is associated with 1 metabolites (arctigenin), 1 substrates (Arctiin), and 1 gut microbiota (*Bifidobacterium longum*).

We constructed the M-S-M-T network to systematically characterize the regulatory framework linking gut microbiota, metabolites, and core target genes, which facilitated the identification of key microbial taxa, metabolites, and gene targets. The network integrated 61 gut microbiota, 21 substrates, 21 non-toxic metabolites, and 5 characteristic target genes. Different node types are indicated as follows: blue triangles represent gut microbiota; orange rhombuses represent substrates; purple hexagons represent metabolites; green ellipses represent gene targets.

### BTBR mice exhibited core ASD-like behavioral phenotypes

**Figure 6:**
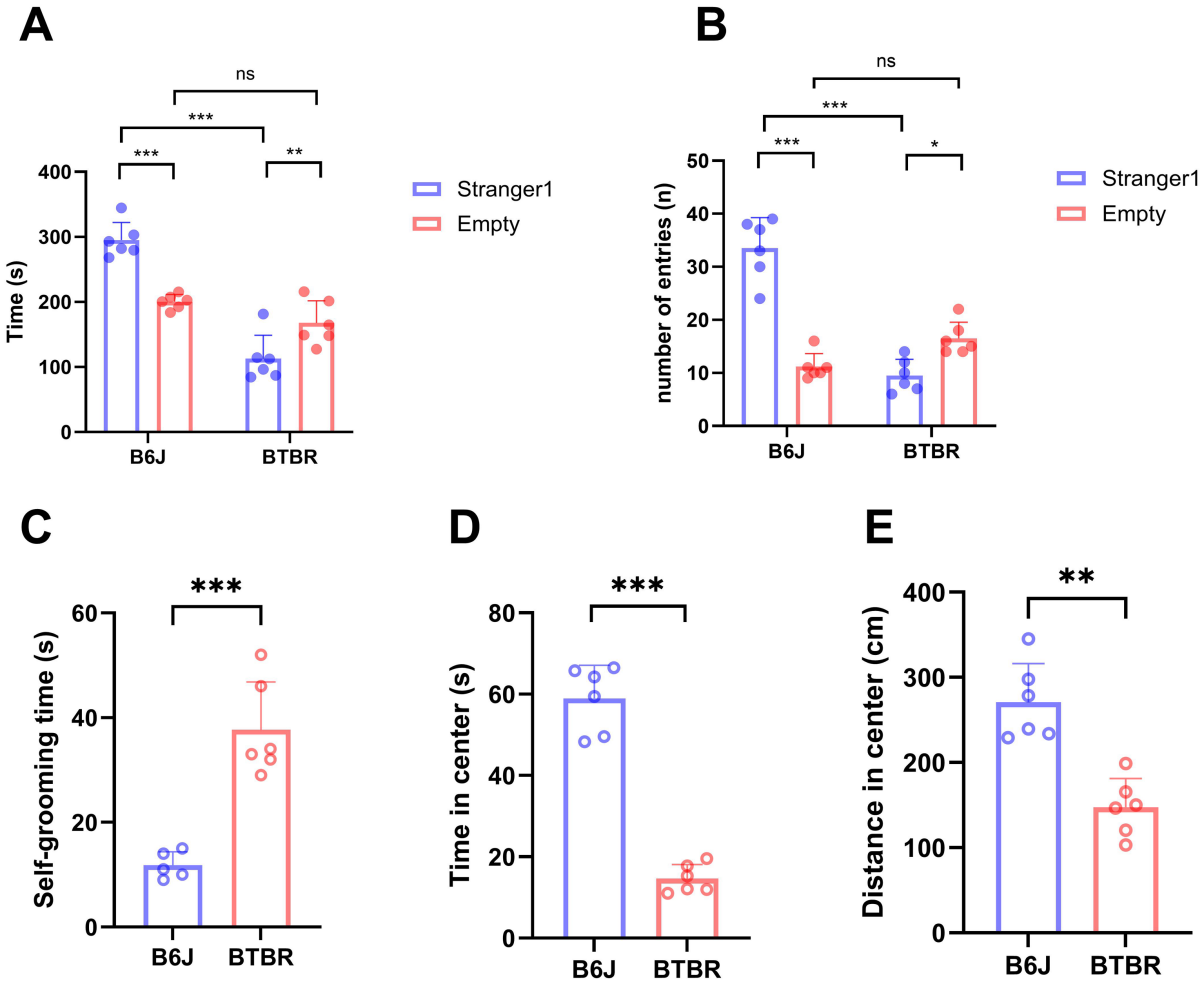
Assessment of social interaction, self-grooming behavior, and open field tests in B6J and BTBR mice. **(A)** Bar graph displaying the time of mice in every group in the chamber with the unfamiliar mouse (Stranger 1) and the empty chamber. **(B)** Bar graph displaying the number of entries of mice in each group in the chamber with the unfamiliar mouse (Stranger 1) and the empty chamber. For quantitative analysis of social behavior, two-way mixed-design repeated-measures ANOVA was used, followed by LSD test for pairwise comparisons within and between groups. **(C)** Bar graph showing the self-grooming time of mice in each group. **(D)** Bar graph displaying the time that mice spent in the central area. **(E)** Bar graph displaying the traveling distance of mice in the central area. For quantitative analysis of self-grooming behavior and open field exploration test, independent samples *t*-test or Welch’s *t*-test was used. The data were presented as means ± SDs. *n* = 6 per group. *p < 0.05, **p < 0.01 and ***p < 0.001.

A series of tests, such as three-chamber social interaction tests, open field and self-grooming behavior, were used to assess the social interaction, and stereotyped and anxiety-associated behaviors of mice. In the three-chamber social interaction test, BTBR mice showed a greater tendency to more frequently enter compartments containing objects, while reducing the frequency and duration of interactions with unfamiliar mice compared to controls. This indicated impaired social approach behavior (*p* < 0.05) (Figures 6A,B). The self-grooming test revealed that BTBR mice exhibited a marked increase in self-grooming behavior (*p* < 0.05) (Figure 6C). The analysis of the open field test data showed a remarkable reduction in entering time and traveling distance relative to the control group. This indicated an increase in anxiety-related behavior (p < 0.05) (Figures 6D,E). These behavioral test results demonstrated a pronounced ASD-like phenotype in BTBR mice.

### Distinct gut metabolic features revealed by metabolomics analysis in BTBR mice

**Figure 7:**
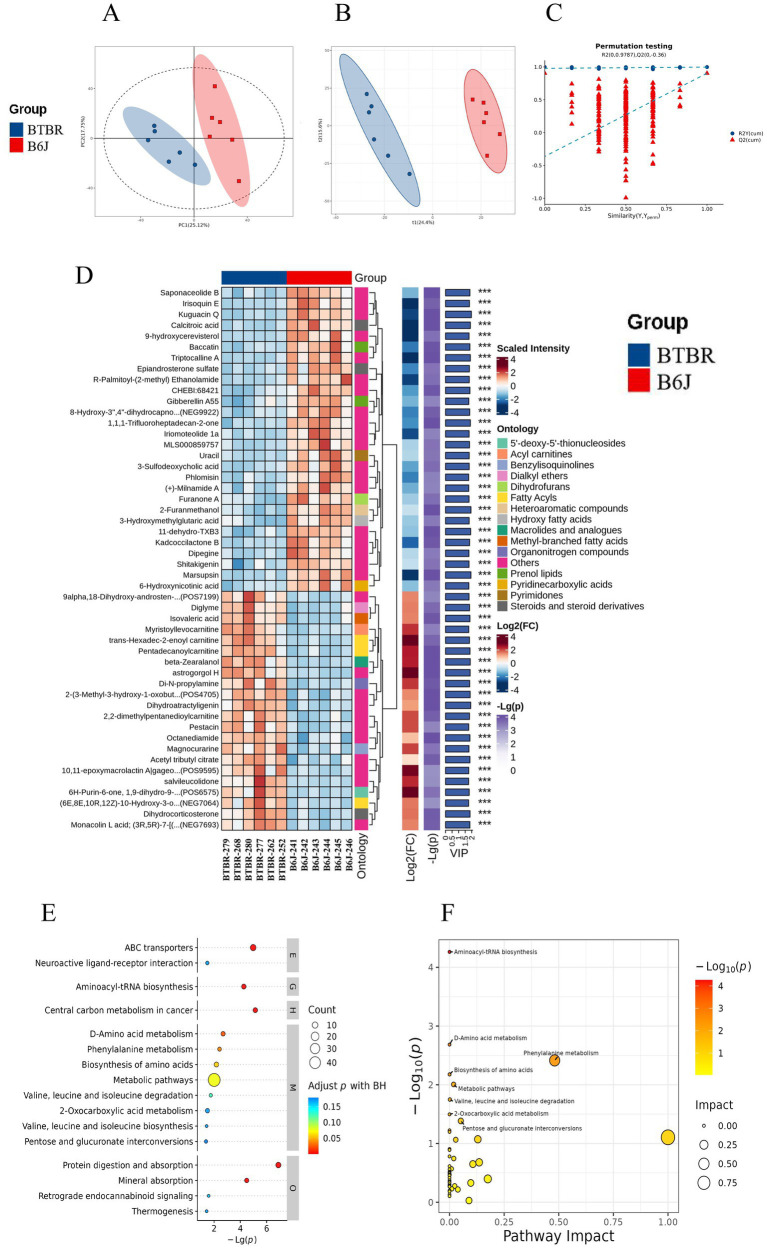
Fecal metabolic profiling and pathway analysis in B6J and BTBR mice. Overview of untargeted fecal metabolomic profiles, including PCA score plots **(A)**, PLS-DA score plots **(B)**, and permutation tests **(C)** to validate model robustness. **(D)** Hierarchical clustering heatmap of significantly differential metabolites between groups (D). KEGG pathway enrichment **(E)** and metabolomic pathway analysis (MetPA) **(F)** of differentially abundant metabolites. *N* = 6 per group.

In this study, non-targeted metabolomics was used to explore changes in the gut metabolites of BTBR mice. The analysis of PCA and PLS-DA demonstrated a marked separation between the two groups (Figures 7A,B). A 200-time random permutation test (RPT) was performed to evaluate the over-fitting of the PLS-DA model. The Q^2^ value was lower than the R^2^ value (Figure 7C). However, this observation alone is insufficient to demonstrate model robustness or exclude potential overfitting. Therefore, the PLS-DA results should be interpreted as exploratory and considered together with the differential metabolite analysis and pathway enrichment results.

In the PLS-DA model, significant differential metabolites were identified based on VIP > 1 and p < 0.05 as the criterion and significance level, respectively. Between control and BTBR mice, primary differential metabolites included organoheterocyclic compounds, lipids and lipid-like molecules, organic acids and derivatives. Clustering analysis was used to select significant differential metabolites between the ASD and control groups. Compared with the control group, multiple metabolites, including Saponaceolide B, Irisoquin E, Kuguacin Q, Calcitroic acid, and 9-hydroxycerevisterol demonstrated a sharp decrease in BTBR mice (Figure 7D). In contrast, a marked increase occurred in metabolites such as Diglyme, Isovaleric acid, 9alpha,18-Dihydroxy-androsten-(4)-dion-(3,17), Myristoyllevocarnitine and trans-Hexadec-2-enoyl carnitine. Comprehensive information on differential metabolites, including retention time, classification, mass-to-charge ratios, *p*-value and additional parameters, is illustrated in Supplementary Table 4. The pathway enrichment analysis of differential metabolites indicated that aminoacyl-tRNA biosynthesis, neuroactive ligand-receptor interaction, D-Amino acid metabolism and phenylalanine metabolism were selected as key metabolic pathways between control and BTBR mice (Figures 7E,F).

### Unique gut microbiotal features revealed by 16S rRNA sequencing analysis in BTBR mice

**Figure 8:**
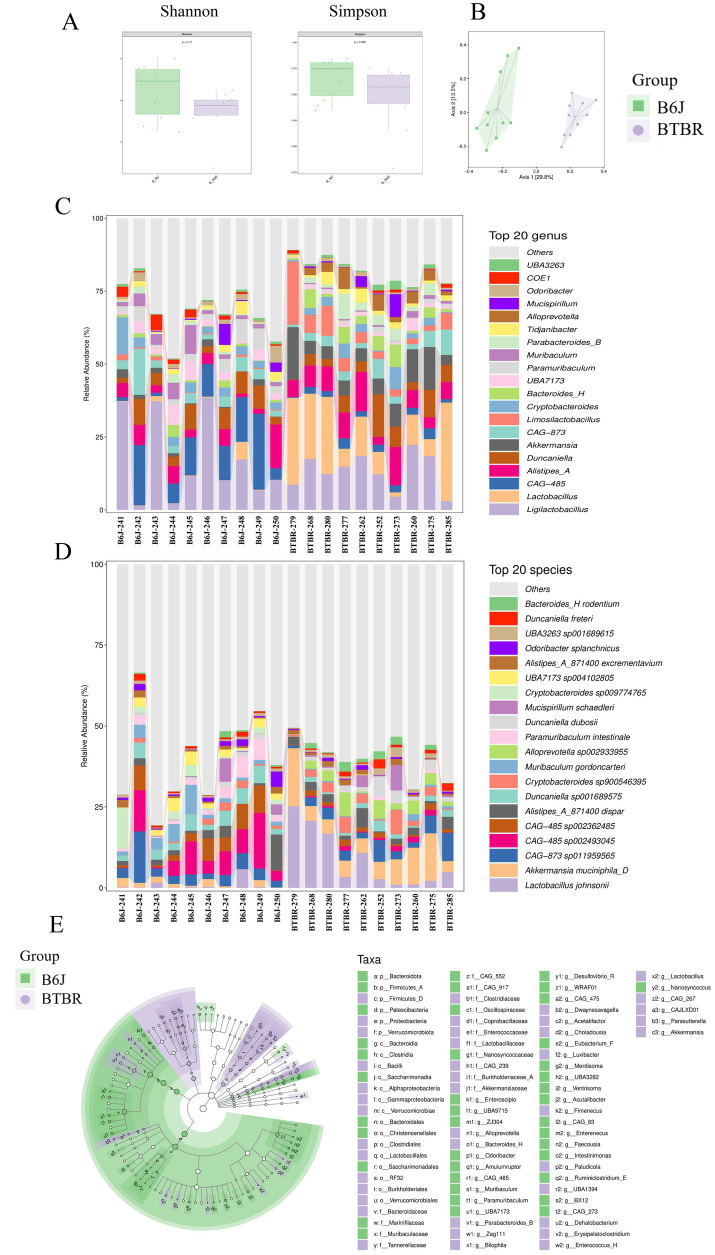
Gut microbiota profiling based on 16S rRNA gene sequencing in B6J and BTBR mice. **(A)** *α*-diversity analysis of gut microbiota based on Shannon and Simpson indices. **(B)** PCoA score plot reflecting *β*-diversity differences between groups. Relative abundance of the top 20 gut microbiota at the genus **(C)** and species **(D)** levels. **(E)** LEfSe analysis revealing differentially abundant taxa between B6J and BTBR groups from phylum to genus level. Green denotes the B6J group, and purple denotes the BTBR group. *n* = 10 per group.

Shannon’s and Simpson’s indices were used to analyze the *α* diversity of gut microbiome samples. Both indexes showed a reduction in BTBR mice compared with control mice, which indicated a decrease in the diversity of their gut microbiome (Figure 8A). The data of *β* diversity were analyzed utilizing principal coordinate analysis (PCoA). The results indicated that both groups showed significant differences in microbial community trends (Figure 8B). These findings revealed that BTBR mice showed a certain degree of alteration in overall gut microbiota structure.

At the genus level, the analysis of relative abundance between both groups suggested that the predominant microbiota in BTBR mice were *Lactobacillus*, *AAkkermansia* and *Alistipes_A* (Figure 8C). At the species level, the relative abundance of both groups was analyzed. The predominant microbiota in the control group were found to be *CAG-485 sp002493045*, *CAG-485 sp002362485* and *Muribaculum gordoncarteri*, which showed a noticeable reduction in BTBR mice (Figure 8D). For further analysis of inter-group species differences, differential bacteria between the two groups were identified using the linear discriminant analysis effect size (LEfSe) method on the basis of the screening criteria of *p* < 0.05 and linear discriminant analysis (LDA) > 2. LEfSe analysis identified 81 candidate differentially abundant genera (*p* < 0.05, LDA > 2), including 40 genera enriched in the control group and 41 enriched in the ASD group (Figure 8E). Given the exploratory nature of LEfSe and the compositional characteristics of microbiome data, these findings should be interpreted with caution and regarded as candidate microbial signatures that warrant further validation in future studies.

The control group showed significantly higher abundance than the ASD group, particularly in *p_Bacteroidota*, *p_Firmicutes_A*, *p_Patescibacteria*, *c_Clostridia*, *c_Bacteroidia*, *c_Saccharimonadia*, *o_Bacteroidales*, *o_Saccharimonadales*, *f_Marinifilaceae*, *f_Muribaculaceae*, *f_Oscillospiraceae*, *f_Nanosyncoccaceae*, *g_ZJ304*, *g_Odoribacter*, *g_Muribaculum*, *g_Paramuribaculum*, *g_UBA7173*, *g_Desulfovibrio_R*, *g_Eubacterium_F*, *g_UBA3282* and *g_Ruminiclostridium_E*.

Higher abundance was identified in the ASD group, including *p_Proteobacteria*, *p_Firmicutes_D*, *c_Alphaproteobacteria*, *c_Bacilli*, *c_Gammaproteobacteria*, *o_Clostridiales*, *o_Lactobacillales*, *o_RF32*, *o_Burkholderiales*, *f_Tannerellaceae*, *f_Bacteroidaceae*, *f_Clostridiaceae*, *f_Coprobacillaceae*, *f_Lactobacillaceae*, *f_Burkholderiaceae_A*, *g_Alloprevotella*, *g_Parabacteroides_B*, *g_Bacteroides_H*, *g_Dwaynesavagella*, *g_Acetatifactor*, *g_Luxibacter*, *g_Fimenecus*, *g_Paludicola*, *g_Dehalobacterium*, *g_Erysipelatoclostridium*, *g_Lactobacillus* and *g_CAJLXD01*. These results suggest that the gut microbiota composition of BTBR mice has undergone significant alterations.

### Exploratory spearman correlation analysis among behavioral performance, differential metabolites, and candidate gut microbiota

In the analysis of correlation between behavioral performance and differentially abundant metabolites, 9-hydroxycerevisterol, Triptocalline A and Irisoquin E (*p* < 0.05) were significantly positively related to social behavior (social time and number of entries into Stranger 1) (Supplementary Figure 3A). In contrast, Diglyme and Isovaleric acid (p < 0.05) showed an inverse relationship with social behavior (social time and number of entries into Stranger 1). In the analysis of correlation between behavioral performance and genus-level differences in the gut microbiota, *g_Odoribacter*, *g_Paramuribaculum*, *g_UBA3282*, *g_UBA7173*, *g_Muribaculum*, *g_CAG-485* and *g_Nanosyncoccus* (*p* < 0.05) were significantly positively linked to social behavior, and negatively related to self-grooming behavior (Supplementary Figure 3B). In the analysis of correlation between differential metabolites and differential gut microbiota at the genus level, Triptocalline A showed a significant positive relationship with *g_Muribaculum* and *g_Odoribacter* (p < 0.05) (Supplementary Figure 3C). A marked positive correlation was noted between CHEBl:68421 and *g_Nanosyncoccus* (*p* < 0.01). Moreover, Baccatin was significantly positively correlated with *g_CAG-485* (p < 0.01). Nevertheless, Irisoquin E demonstrated a significant negative correlation with *g_Alloprevotella*, *g_Bacteroides_H* and *g_Parabacteroides_B* (p < 0.05).

Although these correlations provide supportive evidence for potential interactions among gut microbiota, metabolites, and behavioral phenotypes, they should not be interpreted as evidence of causal or regulatory relationships.

### The expression changes of characteristic genes in BTBR mice were consistent with the results of bioinformatics analysis

**Figure 9:**
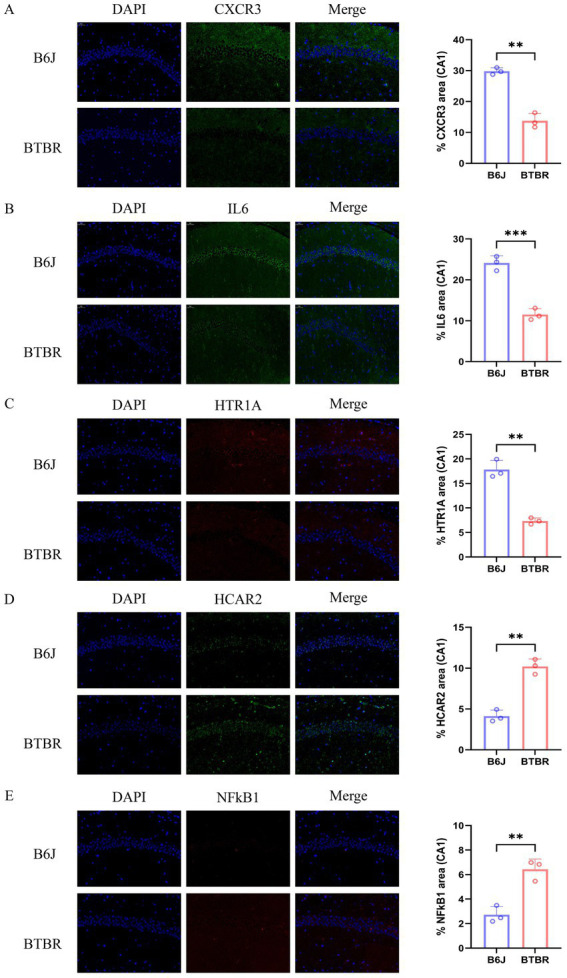
Immunofluorescence analysis of characteristic gene protein expression in the hippocampus of B6J and BTBR mice. **(A–E)** Immunofluorescence staining and quantification of CXCR3, IL-6, HTR1A, HCAR2, and NFKB1 in the hippocampus region of B6J and BTBR mice (scale bar, 50 μm). % Area = (Thresholded positive area / Total image area) × 100%. Quantitative analysis was performed using independent samples *t*-test. The data were presented as means ± SDs. *n* = 3 per group (total 2 field of views (FOV) per mouse, 6 FOVs per group). *p < 0.05, **p < 0.01 and ***p < 0.001.

**Figure 10:**
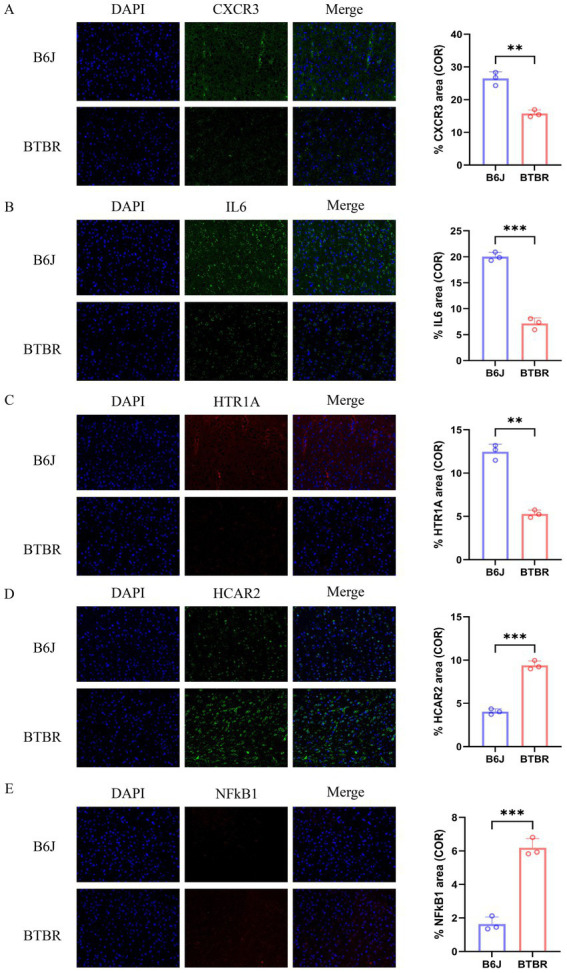
Immunofluorescence analysis of characteristic gene protein expression in the cortex of B6J and BTBR mice. **(A–E)** Immunofluorescence staining and quantification of CXCR3, IL-6, HTR1A, HCAR2, and NFKB1 in the cortical region of B6J and BTBR mice (scale bar, 50 μm). % Area = (Thresholded positive area / Total image area) × 100%. Quantitative analysis was performed using independent samples *t*-test. The data were presented as means ± SDs. *n* = 3 per group (total 2 field of views (FOV) per mouse, 6 FOVs per group). *p < 0.05, **p < 0.01 and ***p < 0.001.

**Figure 11:**
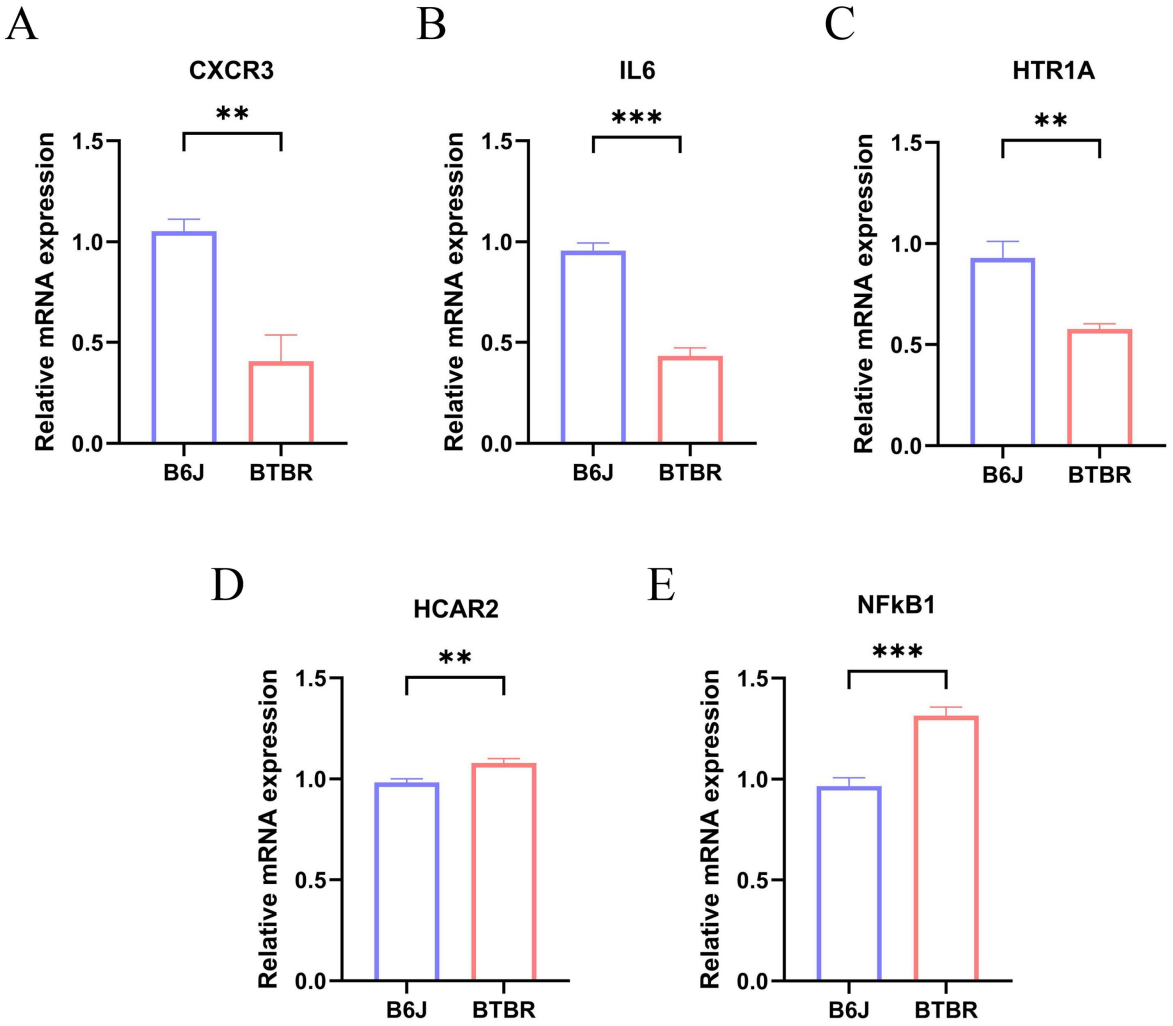
Comparison of hippocampal and cortical mRNA expression levels of characteristic genes in B6J and BTBR mice. **(A–E)** The mRNA expression levels of CXCR3, IL-6, HTR1A, HCAR2, and NFKB1 in the hippocampus and cortex between B6J and BTBR groups. Quantitative analysis was performed using independent samples *t*-test. The data were presented as means ± SDs. *n* = 3 per group. *p < 0.05, **p < 0.01 and ***p < 0.001.

In cortical and hippocampal regions, the five selected characteristic genes were analyzed via immunofluorescence techniques. Compared with control mice, ASD ones showed a drastic up-regulation in the expression levels of HCAR2 and NFKB1, but a substantial reduction in the expression levels of CXCR3, IL6 and HTR1A in both hippocampus (Figures 9A–E) and cortical regions (Figures 10A–E). The RT-qPCR results provided further evidence. Specifically, compared with the B6J group, the mRNA expression levels of HCAR2 and NFKB1 in the cortex and hippocampus of BTBR mice were significantly up-regulated, while the mRNA expression levels of CXCR3, IL-6, and HTR1A were significantly down-regulated (Figures 11A–E) (p < 0.05). These results further confirmed previous bioinformatics analysis results.

## Discussion

Recent research has increasingly focused on the contributions of gut dysbiosis and metabolic disorders to ASD. Alterations in the gut microenvironment may exhibit potential associations with key genes regulating ASD and are closely associated with behavioral manifestations, including social deficits. Consequently, integrating research methodologies such as computational frameworks, machine learning, and network pharmacology can help elucidate gut-brain axis interactions in ASD. For instance, Mohammad et al. (2022) used a computational framework to construct a gut model of host-bacteria interactions, highlighting the dynamic interplay between gut physiology and neural circuits. Olaguez-Gonzalez et al. (2023) applied machine learning algorithms to investigate gut microbiome composition and its potential role in the early diagnosis of ASD, reinforcing the translational potential of the gut microbiome. Tipu et al. reported that probiotic treatment in autistic mice markedly regulates behavioral and neuroplasticity-related genes through the gut microbiota, underscoring the importance of the gut-brain axis (Sherwani et al., 2026). Zhang et al. (2025) employed network pharmacology and multi-database analysis to demonstrate that gut microbiota-derived metabolitesmay serve as key modulators of ASD pathogenesis. Through a multi-omics analysis strategy, Liang et al. (2025) provided preliminary validation of the regulatory role of gut metabolites in disease and emphasized critical associations between gut metabolites and disease phenotypes. Nevertheless, no studies to date have deeply explored the gut-brain communication in ASD by integrating network pharmacology and multi-omics analysis strategies. Therefore, the present study integrates network pharmacology, machine learning and multi-omics to elucidate the gut-brain axis alterations in ASD. Through the combination of network pharmacology and multiple machine learning approaches, CXCR3, HCAR2, HTR1A, IL-6 and NFKB1 were systematically identified as key molecular targets. These targets were validated via immunofluorescence assays. KEGG pathway enrichment analysis revealed that these key targets participate in a range of pathways related to inflammation and metabolism. Notably, these pathways include TLR signaling, linoleic acid metabolism, tyrosine metabolism and cytokine-cytokine receptor interaction. Furthermore, an M-S-M-T network was constructed, which illustrated that core molecules were regulated by gut microbiota-derived metabolites. In addition, untargeted metabolomics and 16S rRNA sequencing analyses were utilized to identify potential correlations among metabolites, brain function and the gut microbiota. Collectively, integrative multi-omics approaches were employed to construct both M-S-M-T and gut microbiota–metabolite–behavior networks, which facilitated the identification of key targets, metabolites and microbial taxa. These findings provide precious insights into the prospective mechanisms of gut–brain communication implicated in ASD progression.

The findings of this study suggest that CXCR3, HCAR2, HTR1A, IL-6 and NFKB1 represent potential core targets implicated in ASD. They were further substantiated through immunofluorescence assays and RT-qPCR analysis. CXCR3 as a G protein-coupled receptor primarily modulates inflammatory and immune responses by binding to chemokines such as C-X-C motif chemokine ligand 9 (CXCL9), CXCL10 and CXCL11. Investigations into the connection between CXCR3 and ASD remain limited. Nevertheless, current evidence indicates that CXCR3 may contribute to the exacerbation of neuroinflammation by facilitating microglial activation or Th1 cell infiltration (Sen et al., 2020). Additional studies have demonstrated that CXCR3 takes part in peripheral immune mechanisms, and the dysregulated activation of the CXCL10/CXCR3 signaling axis can induce long-term effects on hippocampal dendritic architecture (Satrom et al., 2018). Conversely, diminished CXCR3 expression may compromise the capacity of immune cells to eliminate aberrant neural circuits. This results in disrupted synaptic plasticity and consequently influences neurodevelopmental processes and behavioral abnormalities (Blank et al., 2016; Zimmermann et al., 2017). As a multifunctional cytokine, IL-6 is integral to immune regulation and neurodevelopmental processes. Current research demonstrates that IL-6 facilitates microglial activation via the Janus kinase (JAK)/signal transducer and activator of transcription (STAT3) signaling pathway. It also modulates central nervous system inflammation by affecting the balance between Th17 and regulatory T (Treg) cells (Nadeem et al., 2020). Additionally, exposure to exogenous pathogens can induce the expression of IL-6, which subsequently triggers neuroinflammatory responses and disrupts neurodevelopmental processes (Majerczyk et al., 2022). The transcription factor NF-κB1 is pivotal in ASD pathophysiology by regulating hippocampal apoptosis and inflammatory responses (Zhao et al., 2025). HCAR2 contributes to modulating hippocampal neuronal activity and synaptic plasticity through its regulatory effects on lipid metabolism (Zampieri et al., 2025). HTR1A is implicated in neuronal development and emotional regulation in ASD by modulating serotonin (5-HT) signaling pathways (Pourhamzeh et al., 2022). These five targets may not function independently but form a synergistic regulatory network underlying ASD pathogenesis. CXCR3 and IL-6 mediate neuroinflammation and immune cell infiltration; NFKB1 acts as a central hub that transduces inflammatory signals; HCAR2 links lipid metabolism and neuronal function; and HTR1A modulates serotonin signaling and emotional behavior. Together, they bridge peripheral immune disorders, metabolic disturbances, and central nervous system dysfunction, which provides a unique molecular framework for understanding ASD-related pathological changes.

The optimal diagnostic value of CXCR3 was utilized, and its potential roles in ASD were further investigated through single-gene SEA. The cohort that exhibited high CXCR3 expression demonstrated significant enrichment in pathways including focal adhesion, tyrosine metabolism, linoleic acid metabolism, neuroactive ligand receptor interaction, cytokine-cytokine receptor interaction, etc. Conversely, the group exhibiting low CXCR3 expression showed predominant enrichment in pathways such as B cell, NOD-like and TLR signaling pathways, oxidative phosphorylation, and ubiquitin-mediated proteolysis. It has been extensively reported that these pathways play an important part in neuroinflammation and immune dysregulation associated with ASD. Previous research has demonstrated that the prolonged activation of NOD-like and TLR signaling pathways can promote the extensive production of the NOD-like receptor family pyrin domain containing 3‌(NLRP3) inflammasome and pro-inflammatory cytokines. Hence, this aggravates ASD-related neurodevelopmental deficits (Shahi et al., 2025; Aboul-Fotouh et al., 2025). Meanwhile, disruptions in linoleic acid and tyrosine metabolism have been implicated in metabolic imbalances involving molecules like arachidonic acid and dopamine, respectively. This may contribute to impairments in synaptic development and neural function in ASD (Yui et al., 2015; DiCarlo and Wallace, 2022). These findings imply that CXCR3 may modulate the pathogenesis of ASD through its involvement in the aforementioned metabolic and inflammatory pathways, which ultimately affects disease progression. In order to more deeply expound the particular function of CXCR3 within the immune microenvironment, immune cell infiltration and correlation were analyzed across multiple immune cell subsets in ASD individuals. The findings demonstrated that signature genes showed notable synergistic interactions with immune cells. Effector memory CD4 + T, activated CD4 + T, and immature dendritic cells exhibited significant associations with CXCR3. These results suggested that CXCR3 probably has a regulatory effect on the modulation of immune cell infiltration, which thereby influences ASD initiation and progression.

Afterwards, an M-S-M-T network was constructed to explore candidate associations among gut microbiota, gut microbiota-derived metabolites, and ASD-related targets. The network analysis prioritized coumarin, phenylacetic acid, 3-indolepropionic acid, 5-methoxyindole-3-acetic acid, and 5-hydroxyindole-3-acetic acid as candidate metabolites linked to target genes, with favorable predicted drug-likeness and low predicted toxicity. Previous studies have reported that coumarin produced by Bacteroides and Parabacteroides may attenuate oxidative stress in the hippocampus and improve autistic-like behaviors (Karimi et al., 2023). Additionally, phenylalanine metabolism modulated by Bifidobacterium can produce the downstream metabolite phenylacetic acid. Phenylacetic acid has been reported to be elevated in the urine of individuals with autism, suggesting a potential association with ASD (Timperio et al., 2022). Moreover, evidence suggests that acetic acid, propionic acid, and other short-chain fatty acids may participate in the regulation of neuroinflammation, neuronal health, and behavioral symptoms in ASD (Wang et al., 2020; Jabbari Shiadeh et al., 2025). These findings suggest that gut microbiota-derived metabolites may represent candidate mediators linking gut microbial alterations with ASD-related phenotypes. Importantly, phenylacetic acid and coumarin were not identified among the differential fecal metabolites in the present untargeted metabolomics dataset. Therefore, their relevance in this study is mainly supported by network-based prioritization and previous literature rather than direct evidence of altered abundance in BTBR mice. Future targeted metabolomics studies are needed to quantify these compounds and further evaluate their functional relevance in ASD-like phenotypes.

The findings from the preceding bioinformatics analysis reveal a remarkable interconnection among the gut microbiota, gut microbiota-derived metabolites and ASD-related targets. Of note, the gut microbiota and microbiota-derived metabolites appear to play a critical role in ASD initiation and development. This indicates that researchers should place great emphasis on exploring potential interactions in the microbiota–metabolism–brain axis. Given this, untargeted metabolomics alongside 16S rRNA gene sequencing was conducted on fecal samples obtained from normal and ASD model mice to further elaborate on these relationships. The metabolomic profiling of fecal samples was performed utilizing UPLC-ESI-Q-Orbitrap-MS to identify potential biomarkers. The analysis identified 300 metabolites that were significantly different between the control and ASD groups. The identified metabolites predominantly comprised amino acids and derivatives, prenol lipids, fatty acyls, long-chain fatty acids, and carboxylic acids and derivatives. These metabolites were implicated in key metabolic pathways, including neuroactive ligand-receptor interaction, phenylalanine metabolism, D-Amino acid metabolism, valine, leucine and isoleucine degradation, and 2-Oxocarboxylic acid metabolism. Relative to control mice, ASD ones exhibited a significant elevation in the levels of several differential metabolites such as Enol-phenylpyruvate, Phenylalanine and Phenylacetaldehyde, but a marked reduction in the levels of Anandamide. More than that, the dysregulation of the phenylalanine metabolic pathway was substantiated, which thereby corroborated the findings obtained from the bioinformatics analysis.

Research indicates that disruptions in phenylalanine metabolism may play an essential role in ASD pathogenesis. Normally, phenylalanine functions as a crucial precursor in the biosynthesis of dopamine and undergoes a sequence of enzymatic transformations to yield this neurotransmitter. Dopamine, an essential neurotransmitter, is integral to the regulation of neural signal transmission and brain development. Nonetheless, factors such as genetic predispositions, dietary composition and alterations in the gut microbiota give rise to substantial dysregulation in phenylalanine catabolism and utilization. Due to this dysregulation, neurotoxic metabolites like p-cresol and 3-(3-hydroxyphenyl) propanoic acid will accumulate and disrupt synaptic function and exacerbate ASD symptoms (Guzmán-Salas et al., 2022). In addition to that, this metabolic imbalance promotes oxidative stress. In this process, elevated levels of reactive oxygen species inhibit the activity of phenylalanine hydroxylase, diminish the synthesis of dopamine, and consequently lead to the accumulation of phenylalanine (Xian et al., 2025). Importantly, oxidative stress and metabolic dysregulation may serve as the key mediators linking immune activation to brain network alterations (Zwierko et al., 2026). Oxidative stress can further mediate the initiation and progression of inflammation and the activation of immune cells, particularly monocytes and macrophages, thereby exerting an adverse impact on neurodevelopment (Nour-Eldine et al., 2022). Several clinical investigations have complemented these findings and documented pronounced abnormalities in phenylalanine metabolism within urinary samples from ASD children. This highlights a significant correlation between phenylalanine metabolic profiles and autism severity, which thereby suggests their potential utility as biomarkers (Gevi et al., 2016; Xiong et al., 2016). Moreover, Anandamide is a key metabolite involved in the interaction pathway between neuroactive ligands and receptors. It demonstrates therapeutic potential in addressing ASD-associated neurodevelopmental deficits. Research indicates that Anandamide modulates synapse formation and plasticity via the type 1 cannabinoid receptor, which thereby ameliorates synaptic dysfunction (Wang F. et al., 2025). Anandamide has been demonstrated to enhance social cognition and behavior, and mitigate anxiety-like symptoms by elevating oxytocin levels (Campanale et al., 2025). Evidence from numerous animal model investigations further favors the proposition that Anandamide constitutes a promising therapeutic target for ASD (Zamberletti et al., 2017). Correlation analyses provide additional evidence and reveal that differentially expressed metabolites are significantly correlated with behavioral phenotypes, specifically social interaction and self-grooming behaviors. On the whole, these results suggest that the identified differential metabolites and their corresponding metabolic pathways are critical in ASD pathogenesis and progression.

Changes in gut microbiota structure also show a close association with ASD. In the present study, 16S rRNA gene sequencing was used for examining fecal samples from normal and ASD model groups to assess alterations in the composition of the gut microbiota. The findings indicated that BTBR mice demonstrated a remarkable increase in the relative abundance of *g_Lactobacillus*, *g_Akkermansia*, *g_Alloprevotella*, *g_Bacteroides_H* and *g_Parabacteroides_B* in comparison to control ones. Notably, the abundance alteration of *g_Akkermansia* in ASD has not formed an absolute unified consensus in existing research. Our literature retrieval found that increased abundance of *Akkermansia* is more commonly reported in ASD patients, while only a small number of studies have suggested decreased abundance (Lei et al., 2023; Strati et al., 2017; Alamoudi et al., 2022). This variability may be attributable to considerable differences in genetic backgrounds, dietary habits and medication histories among ASD individuals. In preclinical research, our finding of elevated *Akkermansia* abundance in BTBR ASD model mice is highly consistent with the trend reported in most clinical studies, and is also supported by multiple published studies using the same BTBR model, which reflects the inherent, model-specific characteristic of spontaneous microbial dysbiosis in BTBR mice (McFarlane et al., 2008; Golubeva et al., 2017; Sharon et al., 2019). In our study, increased *g_Akkermansia* abundance in BTBR mice was significantly correlated with key metabolic pathway disturbances and impaired social approach behavior, which is completely consistent with our overall regulatory axis of gut microbiota-metabolite-ASD. It should be noted that the clinical extrapolation of this result still needs further verification in larger clinical cohorts, and the specific regulatory mechanism of *Akkermansia* in ASD needs to be confirmed by subsequent targeted intervention experiments. In a range of psychiatric disorders, *g_Alloprevotella* has been significantly associated with neuroinflammation and alterations in brain tryptophan metabolism. It exhibits a positive correlation with cognitive impairments (Wang et al., 2024; Xu et al., 2025). Studies show that the increased abundance of *g_Bacteroides_H* likely facilitates LPS production. In turn, LPSs compromise intestinal barrier integrity and induce excessive Th17 cell activation (Zhao et al., 2024). Additionally, it has been proven that *Bacteroides* activate the NF-κB pro-inflammatory signaling pathway, transmit pathogenic signals to the brain via a damaged intestinal barrier and elicit neuroinflammatory responses (Alexandrov et al., 2019). It has also been shown that *g_Parabacteroides_B* is positively correlated with adverse socio-emotional neurodevelopmental outcomes and may even provoke depression-like behaviors (Davias et al., 2025; Gomez-Nguyen et al., 2021). Furthermore, the correlation analysis conducted identified significant relationships among differential gut microbiota, differential metabolites and behavioral outcomes. As a consequence, *g_Lactobacillus*, *g_Akkermansia*, *g_Alloprevotella*, *g_Bacteroides_H* and *g_Parabacteroides_B* warrant attention in the development of ASD.

The above metabolomic and gut microbiota analyses highlight the critical roles of phenylalanine metabolism and key gut microbiota in ASD. Furthermore, the correlation analysis results further suggest significant associations among ASD behavioral phenotypes, key gut microbiota, and differential metabolites. *g_Bacteroides_H* plays an important role in host-microbiota interactions, particularly in the regulation of metabolic pathways. Studies have shown that phenylalanine metabolism and arginine metabolism are key functional pathways affected by g_Bacteroides and are closely linked to cognitive function (Zhang et al., 2021). Dysregulation of phenylalanine metabolism can severely impair neurodevelopment and lead to behavioral abnormalities (Ashe et al., 2019). Arginine metabolism participates in the modulation of neurotransmission, synaptic plasticity, and memory function in the brain (He et al., 2024). Therefore, *g_Bacteroides_H* may contribute to neurodevelopmental processes such as synaptic function by regulating phenylalanine and arginine metabolism, ultimately influencing behavioral manifestations in ASD. In addition, *g_Parabacteroides_B* is also potentially involved in the regulation of phenylalanine metabolism. An animal study of depression reported that reducing the abundance of *g_Parabacteroides_B* effectively regulated phenylalanine metabolism and further alleviated depression-like behaviors (Deng et al., 2022). Another study demonstrated a positive correlation between *g_Parabacteroides_B* and phenylalanine levels (Fan et al., 2025). Notably, these key microbiota and differential metabolites also represent important nodes in the M-S-M-T network. Combined with our previous M-S-M-T network analysis, the present metabolomic and gut microbiota data further support the critical links among gut microbiota, metabolism, and ASD-related behavioral phenotypes. Although the present study is observational, the consistent changes in phenylalanine metabolism, key microbial genera, and ASD-related behaviors strongly suggest a potential causal cascade. Gut dysbiosis may lead to abnormal metabolite production, which further promotes metabolic pathway disorder, triggers neurological functional and structural impairments, and ultimately contributes to the occurrence of ASD-like behavioral phenotypes. These associations do not simply represent parallel changes but likely constitute a regulatory axis along the gut-brain axis, which provides rational targets and scientific clues for future causal verification.

The foregoing analysis highlights that microbial dysbiosis and metabolites derived from the gut microbiota have a substantial influence on the development of ASD. While this study offers new insights into the gut-brain axis in ASD, several limitations exist. Firstly, this research used GSE18123-a common ASD transcriptomic resource-as data source, but sample characteristics (age, geography, et al.) and methodological variations (e.g., sequencing platforms) across datasets may lead to inconsistent results, requiring validation with multiple datasets; secondly, although the BTBR mouse model (an idiopathic ASD model with social deficits and repetitive behaviors) was employed in this study, different models (e.g., VPA-induced) recapitulate distinct pathological mechanisms and produce different biological outcomes (metabolites, microbiota). Therefore, our conclusions are limited to the BTBR model. Moreover, owing to the absence of healthy mice possessing a genetic background comparable to that of BTBR mice, only male C57 and BTBR mice were employed for metabolomic and 16S rRNA gene sequencing analyses. This may constrain the generalizability of the differential metabolites and microbiota identified. Our study was also limited by the male-predominant nature of both the human dataset and animal models. Future studies should prioritize sex-balanced cohorts and include both male and female animals to explore potential sexual dimorphism in the gut-brain axis of ASD. On top of this, subsequent research needs to be conducted to further validate the mechanistic relationships of the gut microbiota with metabolites in modulating the pathogenesis of ASD.

In the current study, key gut microbiota, core molecular targets and critical metabolites associated with ASD were comprehensively and deeply investigated from a multi-dimensional and systematic perspective. Multi-omics analyses, including network pharmacology, untargeted metabolomics, 16S rRNA gene sequencing, bioinformatics and machine learning, were utilized. The complex interactions between gut microbiota composition and metabolic profiles in the pathophysiology of ASD were illuminated. Novel insights were proposed for future ASD prevention and management. Gut M-S-M-T and gut microbiota–metabolite–behavior networks were developed to systematically characterize the intricate relationships and interactions underlying ASD and the gut microbiota, which thereby helped better understand the gut-brain axis. The findings of this study highlight the significance of the gut microbiota and their metabolic products as potentially modifiable contributors to ASD onset and progression. They offer a novel conceptual framework to inform future therapeutic strategies.

## Conclusion

A multi-omics strategy was taken to clarify the potential mechanisms of the gut microbiota and its metabolites influencing ASD. The findings identified phenylacetic acid and coumarin as principal gut microbiota-derived metabolites. Five core molecular targets, namely CXCR3, HCAR2, HTR1A, IL-6 and NFKB1 modulated by the gut microbiota and their metabolites, may be new therapeutic targets for ASD. Additionally, animal model experiments uncovered significant associations among ASD-related behavioral phenotypes, changes in the composition of the gut microbiota, and differential metabolite profiles. It is worth noting that ASD pathophysiology could be put down to the involvement of specific gut bacterial genera, such as *g_Alloprevotella*, *g_Bacteroides_H*, *g_Parabacteroides_B,* alongside critical metabolic pathways, including phenylalanine metabolism and neuroactive ligand-receptor interactions. In summary, these preliminary results contribute to a better understanding of the treatment potential of metabolites derived from the gut microbiota and offer useful insights into the communication of the gut-brain axis in ASD.

## Data Availability

The original contributions presented in the study are publicly available. The main data generated or analyzed during this study are included in this manuscript and its additional files and are available from the corresponding author on reasonable request. 16s rRNA sequencing data can be found at: https://www.ncbi.nlm.nih.gov/sra/PRJNA1496513.

## Footnotes

1
https://www.ncbi.nlm.nih.gov/geo/

2
http://bio-computing.hrbmu.edu.cn/gutmgene/#/home

3
https://pubchem.ncbi.nlm.nih.gov/

4
http://www.swisstargetprediction.ch/

5
https://sea.bkslab.org/

6
http://www.genecards.org

7
https://go.drugbank.com/

8
https://db.idrblab.net/ttd/

9
https://cran.r-project.org/web/packages/pROC/index.html

10
https://www.bioconductor.org/packages/release/bioc/html/GSVA.html

11
https://cran.r-project.org/package=psych

12
https://cran.r-project.org/web/packages/ggplot2/index.html

13
http://www.swissadme.ch/

14
https://admetlab3.scbdd.com/
