# Supplementary material for: Research on the role of gut microbiota metabolites in autism by multi-omics and network pharmacology
Source: Front Microbiol. 2026 Aug 4;17:1811435. doi: 10.3389/fmicb.2026.1811435 (PMC13481684; doi:10.3389/fmicb.2026.1811435)
Supplement: Supplementary file 1 [file Supplementary_file_1.docx]

**Supplemental Information**

Supplementary Figure 1 Expression profiles, clustering analysis, and diagnostic performance of the five characteristic genes in ASD.

(A) Boxplot visualization of the expression levels of the five characteristic genes (CXCR3, IL6, HCAR2, HTR1A, and NFKB1). (B) Correlation heatmap illustrating ASD subtype clustering based on the random forest (RF) model constructed with the five-gene signature. (C) Receiver operating characteristic (ROC) analysis of the five-gene-based RF model evaluated by five-fold cross-validation in the GSE18123 dataset.

Supplementary Figure 2 Functional enrichment analysis of the five characteristic genes by gene set enrichment analysis (GSEA).

(A-D) GSEA enrichment plots demonstrating the biological functions and signaling pathways associated with the five characteristic genes (CXCR3, HCAR2, HTR1A, IL-6, and NFKB1).

Supplementary Figure 3 Spearman correlation revealed significant associations among behavioral performance, differential metabolites, and key gut microbiota.

(A) Correlation between ASD-behavioral performance and differentially abundant metabolites. (B) Correlation between ASD-behavioral performance and key gut microbiota. (C) Correlation between differential metabolites and key gut microbiota.
