# Supplementary material for: Research on the role of gut microbiota metabolites in autism by multi-omics and network pharmacology
Source: Front Microbiol. 2026 Aug 4;17:1811435. doi: 10.3389/fmicb.2026.1811435 (PMC13481684; doi:10.3389/fmicb.2026.1811435)
Supplement: Supplementary file 2 [file Table_1.DOCX]

Supplementary Table 1 List of primer sequences utilized in this study

| **Gene** | **Primer** | **Sequence (5'-3')** | **PCR Products** |
| --- | --- | --- | --- |
| GAPDH-Mouse | Forward | CCTCGTCCCGTAGACAAAATG | 249bp |
|  | Reverse | TGAGGTCAATGAAGGGGTCGT |  |
| CXCR3-Mouse | Forward | GCCATGTACCTTGAGGTTAGTGA | 87bp |
|  | Reverse | ATCGTAGGGAGAGGTGCTGT |  |
| HCAR2-Mouse | Forward | TTTGAGTCCCAGATGCACCC | 71bp |
|  | Reverse | ACCCTAGGACGAAGAGCCAT |  |
| HTR1A-Mouse | Forward | TACTCCACTTTCGGCGCTTT | 182bp |
|  | Reverse | GGCTGACCATTCAGGCTCTT |  |
| IL-6-Mouse | Forward | AGTTGCCTTCTTGGGACTGA | 159bp |
|  | Reverse | TCCACGATTTCCCAGAGAAC |  |
| NFkB1-Mouse | Forward | AGGAGCAGGACATGGGATTTC | 296bp |
|  | Reverse | CCAAGTGCGAGGTGTCTGATA |  |

Supplementary Table 2 Evaluation of drug-likeness of key metabolites.

| Metabolite | MW | HBA | HBD | TPSA | MLOGP | Lipinski violations | Bioavailability Score |
| --- | --- | --- | --- | --- | --- | --- | --- |
| 2-hydroxy-3-(5-hydroxy-1H-indol-3-yl)propanoic acid | 221.21 | 4 | 4 | 93.55 | -0.01 | 0 | 0.56 |
| 3-Phenylpropionic acid | 150.17 | 2 | 1 | 37.3 | 1.98 | 0 | 0.85 |
| 5-(3-hydroxyphenyl)pentanoic Acid | 194.23 | 3 | 2 | 57.53 | 1.96 | 0 | 0.85 |
| 3-Indolepropionic acid | 189.21 | 2 | 2 | 53.09 | 1.4 | 0 | 0.85 |
| 5-Methoxyindole-3-acetic acid | 205.21 | 3 | 2 | 62.32 | 0.82 | 0 | 0.85 |
| Coumarin | 146.14 | 2 | 0 | 30.21 | 1.65 | 0 | 0.55 |
| Indole-3-lactic acid | 205.21 | 3 | 3 | 73.32 | 0.55 | 0 | 0.85 |
| Secoisolariciresinol | 362.42 | 6 | 4 | 99.38 | 1.56 | 0 | 0.55 |
| 4-Hydroxybenzoic acid | 138.12 | 3 | 2 | 57.53 | 0.99 | 0 | 0.85 |
| 3-(1H-indol-3-yl)propanoate | 188.2 | 2 | 1 | 55.92 | 1.4 | 0 | 0.85 |
| 3-(3-Hydroxyphenyl)propanoic acid | 166.17 | 3 | 2 | 57.53 | 1.37 | 0 | 0.85 |
| Caffeic Acid | 180.16 | 4 | 3 | 77.76 | 0.7 | 0 | 0.56 |
| Dihydrocaffeic acid | 182.17 | 4 | 3 | 77.76 | 0.79 | 0 | 0.56 |
| Phenylacetic Acid | 136.15 | 2 | 1 | 37.3 | 1.66 | 0 | 0.85 |
| Arctigenin | 372.41 | 6 | 1 | 74.22 | 2.12 | 0 | 0.55 |
| 3-(4-Hydroxyphenyl)propionic acid | 166.17 | 3 | 2 | 57.53 | 1.37 | 0 | 0.85 |
| Lariciresinol | 360.4 | 6 | 3 | 88.38 | 1.17 | 0 | 0.55 |
| 3-Hydroxybenzoic acid | 138.12 | 3 | 2 | 57.53 | 0.99 | 0 | 0.85 |
| 3-Hydroxy-4-methoxybenzenepropanoic acid | 196.2 | 4 | 2 | 66.76 | 1.09 | 0 | 0.85 |
| Tryptamine | 160.22 | 1 | 2 | 41.81 | 1.26 | 0 | 0.55 |
| 5-Hydroxyindole-3-acetic acid | 191.18 | 3 | 3 | 73.32 | 0.53 | 0 | 0.85 |

MW: molecular weight < 500; HBA: hydrogen bond acceptor < 10; HBD: hydrogen bond donor ≤ 5; MLog P: Moriguchi octanol-water partition coefficient ≤ 4.15; Lipinski’sviolations ≤ 1; Bioavailability score > 0.1; TPSA: topological polar surface area < 140.

Supplementary Table 3 Evaluation of toxicity of key metabolites.

| Metabolite | hERG-10um | DILI | H-HT | Neurotoxicity-DI | hERG-10um |
| --- | --- | --- | --- | --- | --- |
| 2-hydroxy-3-(5-hydroxy-1H-indol-3-yl)propanoic acid | 0.146879047 | 0.194997922 | 0.495261073 | 0.21620822 | 0.146879047 |
| 3-Phenylpropionic acid | 0.153766274 | 0.108410582 | 0.395799279 | 0.324454308 | 0.153766274 |
| 5-(3-hydroxyphenyl)pentanoic Acid | 0.226953447 | 0.164740145 | 0.472481161 | 0.235079587 | 0.226953447 |
| 3-Indolepropionic acid | 0.131471395 | 0.471417218 | 0.520401955 | 0.396692812 | 0.131471395 |
| 5-Methoxyindole-3-acetic acid | 0.029421238 | 0.990058005 | 0.614436626 | 0.315264791 | 0.029421238 |
| Coumarin | 0.407067716 | 0.754074872 | 0.467896998 | 0.321034938 | 0.407067716 |
| Indole-3-lactic acid | 0.152129471 | 0.312463313 | 0.501357794 | 0.259489 | 0.152129471 |
| Secoisolariciresinol | 0.514378011 | 0.037181448 | 0.6897282 | 0.136022121 | 0.514378011 |
| 4-Hydroxybenzoic acid | 0.144477472 | 0.363316089 | 0.424388021 | 0.402971476 | 0.144477472 |
| 3-(1H-indol-3-yl)propanoate | 0.131471395 | 0.471417218 | 0.520401955 | 0.396692812 | 0.131471395 |
| 3-(3-Hydroxyphenyl)propanoic acid | 0.134793371 | 0.174724609 | 0.466273338 | 0.260004401 | 0.134793371 |
| Caffeic Acid | 0.137909651 | 0.321752131 | 0.449741334 | 0.092591338 | 0.137909651 |
| Dihydrocaffeic acid | 0.189444557 | 0.296994239 | 0.423106223 | 0.051968277 | 0.189444557 |
| Phenylacetic Acid | 0.042977083 | 0.911298871 | 0.369498819 | 0.120021626 | 0.042977083 |
| Arctigenin | 0.805380046 | 0.450175047 | 0.913845003 | 0.450930029 | 0.805380046 |
| 3-(4-Hydroxyphenyl)propionic acid | 0.151547447 | 0.10270863 | 0.408661842 | 0.272970021 | 0.151547447 |
| Lariciresinol | 0.509888053 | 0.422834247 | 0.726688087 | 0.462589324 | 0.509888053 |
| 3-Hydroxybenzoic acid | 0.145169213 | 0.449245095 | 0.475677013 | 0.428288549 | 0.145169213 |
| 3-Hydroxy-4-methoxybenzenepropanoic acid | 0.120748185 | 0.308223158 | 0.472958624 | 0.211100399 | 0.120748185 |
| Tryptamine | 0.581446826 | 0.153618664 | 0.547654331 | 0.765939713 | 0.581446826 |
| 5-Hydroxyindole-3-acetic acid | 0.028346181 | 0.947999299 | 0.594824791 | 0.167915732 | 0.028346181 |

hERG-10uM refers to the inhibitory activity of a compound against the hERG potassium channel at a concentration of 10 µM (micromoles per liter). DILI stands for Drug-Induced Liver Injury. H-H denotes Human Hepatotoxicity. Neurotoxicity-DI represents neurotoxicity, encompassing developmental neurotoxicity and immunotoxicological neurotoxicity. The classification model of ADMETlab outputs probability values ranging from 0 to 1, where a probability between 0.0 and 0.3 indicates a low risk level.

Supplementary Table 4 Comprehensive information of differential metabolites.

| Rt(min) | Ion mode | m/z | Name | Average  (CON) | Average  (ASD) | p-value |
| --- | --- | --- | --- | --- | --- | --- |
| 9.832 | POS | 439.31937 | Salacenonal | 17847741.6033333 | 26411495.95 | 0.00477462289282973 |
| 8.217 | POS | 435.23569 | Asebotoxin | 6883422.2 | 38209013.0716667 | 0.00384985050096474 |
| 9.135 | POS | 434.29355 | Aspochalasin K | 23963030.9966667 | 5095647.93 | 0.00169145420883457 |
| 10.168 | POS | 118.0864 | 3-Amino-2,2-dimethylpropanoic acid | 431700459.943333 | 918460809.603333 | 0.000715950899809314 |
| 14.187 | POS | 431.34976 | Cerevisterol | 43980455.7233333 | 93313012.1833333 | 0.00316640659185376 |
| 8.689 | POS | 431.31049 | 1,2-Dihydro,27-nor-(16beta,22E)-16,18,20-Trihydroxy-24-methylcholesta-1,4,22-trien-3-one | 326498903.093333 | 114801448.378333 | 0.00276961041548175 |
| 11.262 | POS | 429.3345 | Variecoacetal B | 6607385.82333333 | 20698227.4283333 | 0.00907261041551976 |
| 8.871 | POS | 426.35565 | Oleoylcarnitine | 52216797.1083333 | 643847818.698333 | 0.00275592367569267 |
| 11.997 | POS | 425.30371 | 9(11)-dehydroaxinysterol | 6772933.65 | 20105246.79 | 0.00237619152718566 |
| 8.512 | POS | 424.34119 | Linoleoylcarnitine | 12642890.93 | 66901837.6516667 | 0.00304712126490761 |
| 9.033 | POS | 419.24057 | 10,11-epoxymacrolactin A\|gageomacrolactin 1 | 4748263.825 | 35303024.1433333 | 0.000752648619865369 |
| 10.27 | POS | 418.32993 | Olvanil | 497981.168333333 | 6047017.43166667 | 0.00648977650910555 |
| 12.03 | POS | 418.27347 | cespitulactam B | 847386.254166667 | 6842641.7 | 0.000808783493408488 |
| 9.405 | POS | 417.2988 | 12beta,22-Dihydroxy-24-methylscalar-17-en-24,25-olide | 5284956.38833333 | 24987844.0566667 | 0.00187318189088499 |
| 8.972 | POS | 414.35709 | Heptadecanoyl carnitine | 10891298.5483333 | 93927277.8283333 | 0.00589629727169734 |
| 1.247 | POS | 117.05462 | 4-hydroxy-2-methylbut-2-enoic acid | 350527029.711667 | 227389575.473333 | 0.00187472354002005 |
| 7.587 | POS | 407.2778 | 3a,6b,7b,12a-Tetrahydroxy-5b-cholanoic acid | 66959495.6316667 | 136991690.101667 | 0.00465141947069502 |
| 10.136 | POS | 403.23712 | Ambiguine I isonitrile | 35427909.37 | 77877500.9016667 | 0.0048542940442216 |
| 9.609 | POS | 403.23193 | Acetyl tributyl citrate | 20840259.9783333 | 30402050.9433333 | 0.000154084816162406 |
| 8.73 | POS | 400.34036 | L-Palmitoylcarnitine | 91721676.0133333 | 640582715.118333 | 0.00219921751568795 |
| 8.365 | POS | 398.32593 | trans-Hexadec-2-enoyl carnitine | 5371081.28833333 | 59418657.2016667 | 0.000194174392474733 |
| 5.8 | POS | 397.17807 | 2-(3,5-dimethyl-7-oxo-7H-furo[3,2-g]chromen-6-yl)-N-(3-(2-oxopyrrolidin-1-yl)propyl)acetamide | 19871018.4666667 | 80797668.47 | 0.00531040375870335 |
| 10.47 | POS | 395.32959 | 14-Dehydroergosterol | 9746762.985 | 1901138.21 | 0.0130852851023578 |
| 2.675 | POS | 389.13348 | 6-ethyl-3-(4-isopropoxyphenyl)-4-oxo-4H-chromen-7-yl acetate | 25111485.7483333 | 86201971.8033333 | 0.00156997853788135 |
| 8.444 | POS | 386.32486 | Pentadecanoylcarnitine | 7845130.53166667 | 41705309.9733333 | 7.41087584959144e-05 |
| 7.6 | POS | 385.23611 | Dehydrocholic acid | 7222355.22 | 22176269.38 | 0.00172005634888962 |
| 6.065 | POS | 385.21072 | Isorhynchophylline | 17883236.7966667 | 315175390.053333 | 0.00117469021109006 |
| 8.311 | POS | 378.26224 | Prostamide-E2 | 15262969.6966667 | 54777529.29 | 0.00403679627147254 |
| 8.189 | POS | 372.31033 | Myristoyllevocarnitine | 16115731.8016667 | 74108127.6083333 | 0.000437753130315106 |
| 9.028 | POS | 371.2572 | 3-Oxo-4,6-choladienoic acid | 17457723.7816667 | 55031300.13 | 0.00756057105770006 |
| 5.282 | POS | 366.20215 | Ser Ile Phe | 18621401.64 | 45253850.6516667 | 0.00716905307038324 |
| 8.85 | POS | 365.26721 | Sarcotol acetate | 9495987.28333333 | 25050923.1416667 | 0.00826408509243367 |
| 9.488 | POS | 365.267 | 10-Deacetoxybarbilycopodin | 37690813.3016667 | 111582578.263333 | 0.00324253162246746 |
| 0.891 | POS | 365.10419 | Melibiose | 273674820.1 | 104421272.876667 | 0.00316817450739823 |
| 1.345 | POS | 113.03446 | Uracil | 6193094532.70667 | 3355599538 | 0.000315320541442058 |
| 5.366 | POS | 350.20679 | Ala Leu Phe | 11855337.0183333 | 51146448.21 | 0.00768676536187874 |
| 9.488 | POS | 348.28882 | Anandamide | 47328162.9766667 | 24349660.8933333 | 0.00337884956572109 |
| 8.095 | POS | 347.22043 | Cespihypotin P | 1303624.51333333 | 11455030.9566667 | 0.00346061055036871 |
| 4.16 | POS | 346.1637 | o-Methylprecriwelline | 33033744.1833333 | 233165.979166667 | 0.00839261606219881 |
| 4.204 | POS | 343.18652 | 3-HYDROXY-4-(SUCCIN-2-YL)-CARYOLANE delta-LACTONE | 31194273.96 | 85584684.09 | 0.00792005482916483 |
| 1.235 | POS | 75.04462 | Hydroxyacetone | 177402973 | 103728480.5 | 0.00858985240290121 |
| 5.113 | POS | 340.08301 | N-(5,6-dimethoxy-3-oxohydroisobenzofuran-4-yl)(2-methyl(3-furyl))carboxamide | 34272125.42 | 10116130.9283333 | 0.000610363646047588 |
| 8.876 | POS | 337.23645 | Hepoxilin B3 | 35182670.5833333 | 117291800.678333 | 0.00617746091689732 |
| 7.577 | POS | 337.20001 | Dihydrorubrosterone | 19521871.3616667 | 99969336.8166667 | 0.00115515664027781 |
| 6.672 | POS | 333.17941 | Quinocarcinol | 12796558.14 | 1081477.28583333 | 0.00204988863123037 |
| 2.859 | POS | 331.16458 | N-{2-[4-(N,N-diethylcarbamoyl)-2-methoxyphenoxy]ethyl}acetamide | 112802757.471667 | 311053393.126667 | 0.00341661362416331 |
| 7.905 | POS | 330.22623 | 6-Keto-decanoylcarnitine | 380272131.183333 | 630259615.4 | 0.00403182174954565 |
| 7.715 | POS | 326.21375 | farnesyl cysteine | 26026853.7466667 | 45257919.48 | 0.00371935316046519 |
| 4.355 | POS | 326.03748 | Urothion | 23409017.3433333 | 49643732.2316667 | 0.00606783369001325 |
| 9.547 | POS | 324.2894 | Linoleoyl ethanolamide | 3985047769.50333 | 1925725258 | 0.00232263583200382 |
| 8.088 | POS | 323.22055 | Dihydroatractyligenin | 28810308.7766667 | 68878380.37 | 4.04894665948987e-05 |
| 7.164 | POS | 323.18475 | beta-Zearalanol | 11288813.0616667 | 58591235.44 | 7.19932725146411e-05 |
| 9.085 | POS | 322.27386 | Alpha-Linolenoyl ethanolamide | 228391060.3 | 91443507.1933333 | 0.000859293258735483 |
| 7.616 | POS | 319.19006 | 9alpha,18-Dihydroxy-androsten-(4)-dion-(3,17) | 27029226.39 | 74890422.6333333 | 0.000537062057171464 |
| 7.111 | POS | 318.27878 | L 657398 | 24984539.1616667 | 14643333.69 | 0.00225496390279251 |
| 3.993 | POS | 315.18097 | Magnocurarine | 12173336.9 | 50343825.0783333 | 0.000318469937104353 |
| 8.532 | POS | 314.30447 | R-Palmitoyl-(2-methyl) Ethanolamide | 581504204.633333 | 83225711.6666667 | 0.000254814181143638 |
| 4.566 | POS | 314.19382 | Heliotrine | 4389117.02 | 24856562.5866667 | 0.0017228868408863 |
| 5.277 | POS | 313.15411 | Phenylalanylphenylalanine | 10030001.82 | 34615448.93 | 0.00120168593009315 |
| 5.894 | POS | 313.10641 | 1-[2-(2H-1,3-benzodioxol-5-yl)-1-benzofuran-5-yl]propane-1,3-diol | 19447754.6633333 | 5092732.09 | 0.00256440231848106 |
| 11.894 | POS | 310.30988 | N-Hexadecanoylpyrrolidine | 41534472370.6917 | 27752836742.3033 | 0.00456611925552906 |
| 4.667 | POS | 310.1463 | SB 218655 | 83036292.9566667 | 44476217.83 | 0.00760656149259237 |
| 4.719 | POS | 309.18079 | Baquiloprim | 43856496.44 | 4927531.74666667 | 0.00789930087314623 |
| 7.841 | POS | 306.18204 | JWH-167 | 4332930.3 | 258988.968333333 | 0.0102526921565835 |
| 4.066 | POS | 304.17609 | 2,2-dimethylpentanedioylcarnitine | 50070771.9 | 197262588.261667 | 0.000157869426374477 |
| 7.995 | POS | 303.25262 | Glycerol 1-myristate | 55916184.3033333 | 17082184.83 | 0.0097554655072321 |
| 1.764 | POS | 107.04934 | Benzaldehyde | 186562491.888333 | 398592011.448333 | 0.00658058225430705 |
| 4.942 | POS | 302.19586 | (-)-Floridinine | 88110023.3983333 | 4941881.835 | 0.0114018499834966 |
| 4.329 | POS | 299.07996 | 6H-Purin-6-one, 1,9-dihydro-9-(5-S-methyl-5-thiopentofuranosyl)- | 7741136.65 | 67879696.7166667 | 0.000276156009904974 |
| 9.273 | POS | 298.27393 | 3-Ketosphingosine | 1803134346.73167 | 1117486909 | 0.00602178336338109 |
| 4.697 | POS | 297.18085 | 3beta,4alpha,13alpha-Trihydroxylupanine | 39814944.55 | 2097743.105 | 0.0125277138446507 |
| 10.029 | POS | 295.2627 | Methyl linoleate | 229831073.621667 | 703141816.6 | 0.00267517477389334 |
| 11.897 | POS | 293.28308 | SCHEMBL8161733 | 132548844.9 | 84449313.8733333 | 0.00534360043196607 |
| 8.025 | POS | 286.2735 | D-erythro-Sphingosine C-17 | 732662833.843333 | 210086507.191667 | 0.00351695923501781 |
| 10.863 | POS | 285.28812 | Dimethylaminopropyl Lauramide | 184349017.611667 | 119637549.978333 | 0.000592871599518905 |
| 4.213 | POS | 283.16522 | Diethylstilbestrol monomethyl ether | 30942808.1683333 | 3055277.30833333 | 0.00771190325925689 |
| 10.79 | POS | 282.27823 | Oleamide | 148104701985.203 | 102525025460.345 | 0.000896529519445233 |
| 10.185 | POS | 280.26321 | Linoleamide | 216125593326.638 | 135241832931.637 | 0.00215713828786616 |
| 5.9 | POS | 280.18979 | Tramadol N-Oxide | 8042410.08166667 | 18278822.4766667 | 0.00566832501883173 |
| 6.924 | POS | 279.23065 | Hedaol B | 51244018.8666667 | 29347532.8983333 | 0.00516645074162688 |
| 4.384 | POS | 103.07561 | Isovaleric acid | 110144142.056667 | 310872531.241667 | 0.000122426028821148 |
| 5.1 | POS | 279.16949 | H-PHE-LEU-OH | 163585153.998333 | 752076294.251667 | 0.00108287948837698 |
| 8.319 | POS | 278.24759 | Obscuraminol A | 663501008.171667 | 355386855.571667 | 0.0106092223567333 |
| 6.614 | POS | 278.21127 | 10-ethyl-14-methyl-12-oxa-4-azatetracyclo[7.6.1.0?,?.0,?]hexadecan-13-one | 10704379.8166667 | 4502490.9 | 0.0022502054061126 |
| 14.873 | POS | 102.1278 | Di-N-propylamine | 126515885.731667 | 558130809.763333 | 0.000131137316698858 |
| 5.706 | POS | 273.15903 | (E)-5-hydroxy-3-isobutyl-6-(3-methylbenzylidene)-1,6-dihydropyrazin-2(3H)-one | 23801686.9966667 | 13452214.9333333 | 0.00305855548714774 |
| 7.481 | POS | 272.25769 | Hexadecasphingosine | 59405799.3516667 | 36598558.32 | 0.00136783508977394 |
| 8.825 | POS | 271.22647 | 16-Hydroxyhexadecensaure | 29599719.76 | 82296598.26 | 0.00275820705682616 |
| 4.627 | POS | 271.17175 | 17beta-Trenbolone | 53655851.64 | 218480067.616667 | 0.00302438843941525 |
| 4.413 | POS | 271.16461 | SCHEMBL12555514 | 132677645.7 | 3091380.42333333 | 0.0111609691022573 |
| 10.334 | POS | 268.26321 | C17 Sphingosine | 1771373867 | 852964859.3 | 0.00964448906969766 |
| 9.728 | POS | 266.24771 | Lepadiformine B | 163182349.4 | 72491517.5 | 0.00898529726317294 |
| 10.175 | POS | 263.23624 | 6,10,14-Trimethyl-5,9,13-pentadecatrien-2-one | 7749172632 | 4665498656 | 0.0027858619250735 |
| 1.519 | POS | 263.1962 | Exaltolide | 90873063.0033333 | 214928841.546667 | 0.00122690269265144 |
| 9.663 | POS | 261.22086 | Dinortrixagone | 270196512.418333 | 119423254.306667 | 0.00951620578929165 |
| 4.176 | POS | 261.14432 | Glutamylleucine | 339502279.2 | 762637705 | 0.00827032042001961 |
| 7.072 | POS | 259.09561 | Pestacin | 7853913.69666667 | 30721860.03 | 0.000519403790740833 |
| 10.602 | POS | 256.26315 | Palmitic amide | 81675028969.0933 | 58912633744 | 0.00233693067028677 |
| 6.021 | POS | 255.19508 | 4,7,7,11-Tetramethyl-12-oxabicyclo[9.1.0]dodec-4-ene-3,6-diol | 21908632.91 | 3943367.20833333 | 0.00244558941191467 |
| 7.168 | POS | 253.21591 | Hydnocarpic acid | 17155541.4283333 | 10883071.88 | 0.014012955254028 |
| 5.5 | POS | 252.15877 | 4-methoxytetrahydrosecurinine | 24216968.66 | 41097261.87 | 0.00736031545151987 |
| 4.846 | POS | 248.15135 | 4,6-Dideoxy-3-C-methyl-4-(methuylamino)mannose,9CI-beta-D-Pyranose-form-Me glycoside,N-Ac | 47565562.5966667 | 306622333.858333 | 0.00651426126805168 |
| 6.431 | POS | 248.12759 | Phyllanthine | 52065125.53 | 133348449.251667 | 0.0107880990067877 |
| 4.225 | POS | 247.14413 | Hypaphorine | 12737398.8516667 | 28586990.0933333 | 0.00691833980059468 |
| 6.465 | POS | 247.13234 | Estafiatin | 9066726.48833333 | 18925238.27 | 0.00744998984758377 |
| 4.145 | POS | 246.12323 | 4-Acetamidoantipyrine | 16426011.3733333 | 40523984.7533333 | 0.00479946517422157 |
| 10.171 | POS | 245.22539 | (8R,9R,10S,13S,14S)-13-Methyl-1,4,5,6,7,8,9,10,11,12,14,15,16,17-tetradecahydrocyclopenta[a]phenanthrene | 1286231652 | 784871547.843333 | 0.0025873296146044 |
| 5.111 | POS | 245.1378 | Pandangolide 1a | 257734015.7 | 111164943.588333 | 0.00830152147764772 |
| 0.911 | POS | 244.07832 | N-Acetylmannosamine | 4194827594.76667 | 1604523676.15833 | 0.00236157858609083 |
| 1.245 | POS | 99.04431 | 2-Furanmethanol | 316858689.161667 | 193271121.601667 | 1.9618111658467e-05 |
| 1.258 | POS | 73.02892 | Malondialdehyde | 73347509.5333333 | 39348489.6816667 | 0.00845087807208468 |
| 4.305 | POS | 240.12311 | NCGC00380297-01 | 381898959.3 | 121842182 | 0.0029499707129383 |
| 1.765 | POS | 240.08611 | 2-acetamido-2-[3-hydroxy-4-(hydroxymethyl)phenyl]acetic acid | 171030604.668333 | 92545978.2633333 | 0.00860928983678079 |
| 6.238 | POS | 239.12694 | 2-(3-Methyl-3-hydroxy-1-oxobutyl)-4-(1-hydroxyethyl)phenol | 9287832.435 | 24998935.71 | 8.48073007199967e-05 |
| 10.175 | POS | 238.21608 | (+)-Isoaminobisabolenol a | 221776201.3 | 126893106.591667 | 0.00135053965379589 |
| 9.772 | POS | 237.22096 | Bombykal | 133650807.913333 | 68124879.02 | 0.00514630170920583 |
| 6.017 | POS | 237.18427 | Godotol A | 36236236.4583333 | 5631778.45166667 | 0.000715239207358077 |
| 3.724 | POS | 237.12309 | ALANYL-dl-PHENYLALANINE | 28976098.84 | 68979993.1466667 | 0.00247682507950439 |
| 9.346 | POS | 235.20525 | 4-Ethyl-7,11-dimethyldodeca-trans-2-trans-6-1-o-trien-1-al | 93537225.66 | 38715741.07 | 0.00811572361527425 |
| 4.73 | POS | 231.11259 | (1xi,3xi)-1,2,3,4-Tetrahydro-1-methyl-beta-carboline-3-carboxylic acid | 271475993.666667 | 174806761.8 | 0.00443247022852064 |
| 9.561 | POS | 228.23192 | Halaminol C | 4670869684.52667 | 2329315682.61167 | 0.00516617024498943 |
| 5.124 | POS | 227.12759 | 11-Hydroxyjasmonic acid | 375615166.788333 | 202039660.9 | 0.00487257314362774 |
| 9.019 | POS | 226.21629 | N-(2-Methylpropyl)dec-2-enamide | 544134488.6 | 265138241.6 | 0.00615254234320819 |
| 8.155 | POS | 225.18449 | SCHEMBL7552976 | 41335252.5983333 | 7048311.37666667 | 0.0113644287421513 |
| 1.255 | POS | 97.02869 | Protoanemonin | 188733202.591667 | 96693489.9466667 | 0.0063435286478432 |
| 10.203 | POS | 224.2005 | (E,E)-2,4-Decadienoic isobutylamide | 388893844.076667 | 228581489.551667 | 0.00211144655905424 |
| 1.433 | POS | 224.09161 | Salsolinol-1-carboxylic acid | 43016872.39 | 6442198.41916667 | 0.00509415311215178 |
| 3.804 | POS | 223.14377 | 3-Hydroxymonoethylglycinexylidide | 86763191.8733333 | 4877895.10166667 | 0.00649330095457179 |
| 9.77 | POS | 219.20996 | (Z,E)-3,4,7,11-Tetramethyl-1,3,6,10-dodecatetraene | 52622910.1933333 | 26042104.1833333 | 0.00365831621630217 |
| 6.548 | POS | 216.15891 | Ethyl butylacetylaminopropionate | 68279165.8983333 | 103860241.056667 | 0.0117435561217414 |
| 3.634 | POS | 211.13132 | (R)-(+)-Patulolide B | 9717047.81666667 | 24982177.1233333 | 0.00463043322216806 |
| 10.191 | POS | 210.18459 | Converzin | 382050683.183333 | 229300958.423333 | 0.00280162910846441 |
| 4.353 | POS | 210.11241 | Anhalamine | 863455497.243333 | 521832648.623333 | 0.00474921374184123 |
| 5.12 | POS | 209.11681 | (+)-4,5-Didehydrojasmonic acid | 98650555.2216667 | 50492852.04 | 0.00482539472466715 |
| 3.86 | POS | 208.13284 | Benzoylcholine | 34814760.5066667 | 6154550.45333333 | 0.00285758661316133 |
| 5.424 | POS | 208.09647 | N-Acetyl-DL-phenylalanine | 29938068.1566667 | 57507133.98 | 0.000737823777989202 |
| 2.852 | POS | 207.11232 | 1,2,3,3a,4,9-Hexahydropyrrolo[2,1-b]quinazoline-3,3a-diol | 83755897.7733333 | 152623458.7 | 0.00199562790881537 |
| 3.798 | POS | 201.14697 | UNII-UOM92D5748 | 23628903.31 | 70617141.5066667 | 0.0106893477175748 |
| 8.693 | POS | 200.20012 | Dodecanamide | 1224869575 | 771040018.1 | 0.00272969619599733 |
| 10.203 | POS | 198.18462 | Cyclododecalactam | 720575087.9 | 418279039.9 | 0.00198037624458365 |
| 1.146 | POS | 198.09723 | D-Glucose | 417313318.5 | 210999579.268333 | 0.00357778718632741 |
| 10.194 | POS | 196.16939 | dodeca-2,4-dienamide | 274332054.968333 | 168853266.071667 | 0.00626005341362982 |
| 9.071 | POS | 195.17365 | Geranylacetone | 8727778.70333333 | 33538417.3366667 | 0.000494009447730957 |
| 6.465 | POS | 193.08542 | 5-[Hydroxy(phenyl)methyl]dihydrofuran-2(3H)-one | 8991290.2 | 17765381.6466667 | 0.00754099299944483 |
| 5.108 | POS | 191.10651 | Schiffnerone B | 92055359.47 | 35442560.16 | 0.00143502300508229 |
| 1.001 | POS | 187.05746 | Rhamnose | 301365372.841667 | 117517373.551667 | 0.00493617224876676 |
| 8.298 | POS | 186.18488 | N-undecan-2-ylidenehydroxylamine | 291317365.918333 | 160529867 | 0.00910823470459078 |
| 10.602 | POS | 72.04449 | Acrylamide | 142577836.7 | 102836553.403333 | 0.00633890017814858 |
| 10.205 | POS | 184.16922 | Tecostanine | 615351982.6 | 370728627.7 | 0.00261682096925651 |
| 10.194 | POS | 182.1539 | (+)-Myrioxazine B | 199324936.861667 | 117947507.4 | 0.00222557519561919 |
| 4.477 | POS | 180.10187 | Methyl l-phenylalaninate | 53375662.7783333 | 102503059.6 | 0.00933373582545387 |
| 1.189 | POS | 180.08653 | Mannosamine | 179361360.1 | 104703105.3 | 0.00463837898035231 |
| 10.173 | POS | 179.17871 | 1,4,7-Tridecatriene | 109261155 | 63095117.8466667 | 0.00409774054307045 |
| 5.966 | POS | 176.07042 | Indoleacetic acid | 44528719.22 | 76088664.6783333 | 0.00225642693406165 |
| 1.546 | POS | 173.12813 | Octanediamide | 314120209.236667 | 591389892.143333 | 7.19162154399754e-05 |
| 5.424 | POS | 172.13286 | Gabapentin | 2163529025.24833 | 1380903343.38833 | 0.00714732777128313 |
| 10.192 | POS | 170.15341 | Nitramine | 503262756.9 | 295868194 | 0.00174024964021569 |
| 10.184 | POS | 168.13812 | Deca-2,4-Dienamide | 118682851.5 | 71387400.2533333 | 0.0053852795927743 |
| 1.008 | POS | 165.05438 | Enol-phenylpyruvate | 277835941.2 | 441413934.828333 | 0.00946327647228336 |
| 5.116 | POS | 163.11162 | 4,4alpha,5,6-Tetrahydro-7-methyl-2(3H)-naphthalenone | 56746390.45 | 23694271.2483333 | 0.00203811587232974 |
| 1.244 | POS | 163.06 | 3-Hydroxymethylglutaric acid | 173343815.8 | 79065781.94 | 0.00036106878607425 |
| 10.185 | POS | 161.13188 | Trinoranastreptene | 95259604.29 | 51947849.87 | 0.00543155270269037 |
| 1.093 | POS | 157.04674 | 2-(hydroxymethyl)-5-methoxy-4H-pyran-4-one | 123217754.2 | 28945072.41 | 0.00477317583420762 |
| 1.347 | POS | 156.06522 | 7-oxo-1-azabicyclo[3.2.0]heptane-2-carboxylic acid | 248217518.858333 | 161260128.738333 | 0.00216538506805146 |
| 1.251 | POS | 85.02876 | Furanone A | 335586559.9 | 158021292.128333 | 0.000257414207772987 |
| 0.944 | POS | 84.08114 | Tetrahydropyridine | 243980506.411667 | 322440503.661667 | 0.00949754691343656 |
| 4.069 | POS | 146.11734 | L-beta-Homoleucine | 68622016.9283333 | 292212790.403333 | 0.00759206114426092 |
| 10.186 | POS | 142.12219 | (卤)-Pelletierine | 359516225.701667 | 246906589.198333 | 0.00773531694014343 |
| 7.3 | POS | 817.57996 | Hyocholic acid | 75512788.4433333 | 246559806.7 | 0.00806348623722681 |
| 1.419 | POS | 140.03389 | 6-Hydroxynicotinic acid | 290964712.5 | 121477438.068333 | 0.000354235173520202 |
| 4.376 | POS | 135.10159 | Diglyme | 396970452.8 | 1140655101 | 0.000218914347409875 |
| 1.506 | POS | 132.10168 | Isoleucine | 168403107396.208 | 245948142874.76 | 0.00845510374198337 |
| 9.762 | POS | 675.53815 | SM(d16:1/16:0) | 15692714.8433333 | 4841449.15166667 | 0.00484200263254126 |
| 7.896 | POS | 607.41827 | MK 800-62F1 | 25150069.2466667 | 65754530.38 | 0.0127773057197264 |
| 10.174 | POS | 128.10695 | N-Cyclohexylformamide | 280165700.2 | 182402242.021667 | 0.00511153754949996 |
| 11.046 | POS | 518.31982 | 1-Palmitoylphosphatidylcholine | 30561170.61 | 80204602.0883333 | 0.0133922849651465 |
| 7.404 | POS | 491.37091 | Cycloastragenol | 4385511.355 | 20687109.9383333 | 0.00267767959875044 |
| 7.206 | POS | 491.33612 | Phlomisin | 38786672.79 | 9346984.20833333 | 0.00046672528622364 |
| 10.604 | POS | 490.42352 | alpha-Tocopherol acetate | 16832642.97 | 480298.38 | 0.00176680861307177 |
| 9.705 | POS | 479.37161 | Clathriol | 72061412.6966667 | 170643254.201667 | 0.011447682688347 |
| 8.968 | POS | 479.37103 | Dendronesterol A | 26456818.6366667 | 77977390.04 | 0.00747667253573367 |
| 10.446 | POS | 477.35678 | Oogoniol | 29943223.2383333 | 66040334.4883333 | 0.0117829626114019 |
| 5.119 | POS | 121.10113 | Isopropylbenzene | 110672001.936667 | 49288444.7083333 | 0.00443972208970074 |
| 10.923 | POS | 463.37668 | Irisoquin F | 176192135.858333 | 473297798.886667 | 0.0106569130352827 |
| 9.924 | POS | 463.34128 | Polyporusterone F | 9923713.93833333 | 41073543.1983333 | 0.00145504476005163 |
| 2.852 | POS | 120.08091 | 2-Hydroxyphenethylamine | 20064798871.9467 | 34845005375.595 | 0.00206435049524989 |
| 11.262 | POS | 447.34506 | Gibberoketosterol | 19054923.9883333 | 65653862.54 | 0.00452456566974503 |
| 4.852 | NEG | 377.12439 | 8-Hydroxy-3'',4''-dihydrocapnolactone-2',3'-diol | 27647713.7216667 | 6740222.15333333 | 0.00015280465790793 |
| 0.934 | NEG | 377.08475 | D-Maltose | 272197349.676667 | 83721430.35 | 0.0031042847915245 |
| 8.861 | NEG | 373.23773 | Calcitroic acid | 42956346.7583333 | 4961501.18666667 | 4.62873009971027e-05 |
| 8.58 | NEG | 373.20535 | 3alpha-hydroxy-19-acetoxyspongia-13(16),14-dien-2-one | 174704022.556667 | 43862313.6216667 | 0.00361341002033037 |
| 5.093 | NEG | 371.21799 | TMC-96 | 8479515.46666667 | 5126744.99666667 | 0.00491752518517008 |
| 9.09 | NEG | 369.27603 | N-Palmitoyl Asparagine | 5774074.23 | 1567209.12333333 | 0.00458451923258464 |
| 11.391 | NEG | 369.17355 | Epiandrosterone sulfate | 6600025.44333333 | 1708073.295 | 0.000309115589977885 |
| 9.546 | NEG | 368.28033 | (Z)-myristoleoylcarnitine | 136804329.3 | 52812186.0566667 | 0.00592603634375224 |
| 6.464 | NEG | 368.11783 | Porritoxin sulfonic acid | 1494474.72083333 | 10570181.1683333 | 0.00247453339145066 |
| 10.589 | NEG | 367.15799 | Testosterone sulfate | 11186584.4333333 | 5025967.52666667 | 0.0100519484235999 |
| 6.453 | NEG | 365.19705 | 11-dehydro-TXB3 | 35365182.02 | 15183738.4 | 3.24846671724145e-05 |
| 8.846 | NEG | 363.25473 | Ciprostene | 17477165.8483333 | 53014373.38 | 0.00418773690138202 |
| 5.61 | NEG | 363.23895 | JWH 007-d9 | 32526244.4316667 | 6274366.99166667 | 0.00194852526763072 |
| 6.359 | NEG | 363.14539 | Gibberellin A55 | 41856131.15 | 15746205.79 | 0.000513395770216625 |
| 10.841 | NEG | 355.32196 | 13-Hydroxydocosanoic acid | 3268851.10333333 | 1440194.35 | 0.00934648664322871 |
| 10.893 | NEG | 355.15854 | Dipegine | 180919557.248333 | 99938550.9316667 | 0.00038713879298713 |
| 8.614 | NEG | 349.24365 | 2beta,3beta,4beta-Trihydroxypregnan-16-one | 21399155.47 | 72340233.8 | 0.00922861336907572 |
| 8.591 | NEG | 349.23874 | 13,14-dihydroxy-11-mulinen-20-oic acid | 29656438.6466667 | 104850933.748333 | 0.00261235392960841 |
| 5.511 | NEG | 348.14914 | Foliosidine acetate | 8246816.73 | 67237099.28 | 0.00325363162604468 |
| 8.348 | NEG | 347.2225 | Dihydrocorticosterone | 3184405.25666667 | 9965600.53 | 0.000139050455798533 |
| 6.46 | NEG | 347.15298 | 15-acetoxy-1beta,13,14-trihydroxy-8,11,13-podocarpatrien-7-one | 18524352.84 | 1863150.33166667 | 0.0108353193545064 |
| 0.938 | NEG | 347.07492 | 3-Benzyloxy-2-phenyl-benzo[b]thiophen-1,1-dioxid\|3-benzyloxy-2-phenyl-benzo[b]thiophene 1,1-dioxide | 176518391.896667 | 76790011.31 | 0.00969352122772071 |
| 6.155 | NEG | 345.22781 | oscr#20 | 170971265.588333 | 95532447.3666667 | 0.00885326274343717 |
| 10.278 | NEG | 341.14313 | guanacastepene M | 46064847.8233333 | 10582636.96 | 0.00288088433797591 |
| 5.505 | NEG | 337.13254 | DIM-C-pPhOH | 84126544.7966667 | 5266653.9 | 0.0132894436035356 |
| 8.3 | NEG | 335.22348 | (5Z,8Z,10E,12S,14Z,19R)-12,19-Dihydroxy-5,8,10,14-eicosatetraenoic acid\|(5Z,8Z,10E,12S,14Z,19S)-12,19-Dihydroxy-5,8,10,14-eicosatetraenoic acid | 49205802.9333333 | 113067826.133333 | 0.00830337401818228 |
| 5.204 | NEG | 331.17703 | heliconol C | 24270479.6866667 | 15127959.5716667 | 0.00286658188911199 |
| 5.841 | NEG | 329.10257 | Sellowin B | 984849983.8 | 390023923.5 | 0.00238906412185832 |
| 8.9 | NEG | 328.24893 | 4,8 Dimethylnonanoyl carnitine | 567967305.461667 | 269378998.926667 | 0.0010529876664982 |
| 5.639 | NEG | 327.08716 | 2,4-bis(4-hydroxyphenyl)cyclobutane-1,3-dicarboxylic Acid | 122842650.598333 | 28803099.5033333 | 0.0116963798133429 |
| 8.624 | NEG | 323.22272 | TOFA | 98374201.4183333 | 11953025.8416667 | 0.00160003597927932 |
| 7.075 | NEG | 323.18668 | Hynapene A | 16506501.0433333 | 3555687.74666667 | 0.00759765807715208 |
| 8.09 | NEG | 321.20694 | Monacolin L acid; (3R,5R)-7-[(1S,2S,6R,8aR)-2,6-Dimethyl-1,2,6,7,8,8a-hexahydronaphthalen-1-yl]-3,5-dihydroxyheptanoate | 36146173.6916667 | 102517147.106667 | 0.000149694868625699 |
| 1.745 | NEG | 317.09866 | Evofolin B | 12781623.57 | 31237248.7566667 | 0.00805505307369205 |
| 5.714 | NEG | 315.12424 | Sequosempervirin B | 173449652.698333 | 93677632.9566667 | 0.00285220342604309 |
| 4.961 | NEG | 314.10666 | 2-((2,6-Diethylphenyl)(methoxymethyl)amino)-2-oxo-ethanesulfonic acid | 27166872.9566667 | 14209418.8916667 | 0.00274723412573111 |
| 10.366 | NEG | 310.23828 | N-Tetradecanoyl-DL-homoserine lactone | 35965653.5933333 | 15463939.8233333 | 0.0102353517763104 |
| 10.372 | NEG | 307.22781 | 1,1,1-Trifluoroheptadecan-2-one | 18602722.7116667 | 5187053.25666667 | 6.09906348319645e-05 |
| 8.349 | NEG | 307.19193 | Corchorifatty acid D | 222999013.5 | 13732821.11 | 0.00650437578512542 |
| 7.84 | NEG | 307.19125 | (6E,8E,10R,12Z)-10-Hydroxy-3-oxooctadecatrienoic acid | 4515854.78 | 13599459.84 | 0.000759409810775395 |
| 5.418 | NEG | 301.07135 | Marsupsin | 67197543.8266667 | 8778721.45666667 | 1.39889590468551e-05 |
| 7.832 | NEG | 295.19189 | 12-acetoxy-11-hydroxypseudoguaian-4-one | 11816616.7016667 | 3045229.79666667 | 0.00138706359906001 |
| 5.081 | NEG | 295.12277 | corianin\|dihydrocorianin | 24389166.4333333 | 9901349.79833333 | 0.00108989804934023 |
| 1.457 | NEG | 289.0679 | Epicatechin | 102128949.4 | 29610652.5783333 | 0.00473603012588414 |
| 5.077 | NEG | 287.18652 | 6-Hydroxypentadecanedioic acid | 521546089.351667 | 253414251.666667 | 0.00333814808796489 |
| 4.734 | NEG | 287.14975 | 5-Cx-MEHA | 50660967.0733333 | 22172121.5316667 | 0.00149492593274575 |
| 5.101 | NEG | 287.09253 | ACMC-20n3qw | 2375778.255 | 17537685.4433333 | 0.00946242766572585 |
| 5.85 | NEG | 285.11313 | Diphenolic acid | 99997906.6766667 | 43184296.9733333 | 0.00295530000969768 |
| 11.888 | NEG | 283.26447 | Stearic acid | 8669265.835 | 2111482.10916667 | 0.00409374491027436 |
| 10.928 | NEG | 283.22812 | 12-(2,3-Dihydroxycyclopentyl)-2-dodecanone | 29136790.4366667 | 5646435.855 | 0.00523855262638829 |
| 4.52 | NEG | 283.09399 | MLS000859757 | 5661655.27333333 | 2885029.11666667 | 0.000291460062197337 |
| 7.579 | NEG | 281.17621 | Gallicynoic acid C | 15407912.02 | 4042853.47 | 0.000808046221580059 |
| 12.592 | NEG | 279.11105 | 2,7-Acetylaminofluorene | 31120813.7 | 19482598.0133333 | 0.0105668086036947 |
| 0.962 | NEG | 279.03854 | Uridine | 155574816.923333 | 35697291.9616667 | 0.00222498282676484 |
| 9.238 | NEG | 277.18399 | Tetradecane-7-sulfonic acid | 105125498.4 | 219274922.2 | 0.0010935660668561 |
| 7.555 | NEG | 267.16046 | Arteincultone | 63492011.28 | 20458170.06 | 0.00562152366200799 |
| 1.758 | NEG | 263.04355 | Necatorine | 68650562.4233333 | 35622796.3766667 | 0.00257303242437086 |
| 6.415 | NEG | 257.08176 | 2',4',6'-Trihydroxydihydrochalcone | 14985341.08 | 1286513.685 | 0.00616870597267527 |
| 6.192 | NEG | 591.3205 | Mesobilirubinogen | 26291810.0666667 | 6852603.075 | 0.000971426862767817 |
| 5.608 | NEG | 575.32159 | 2',3'-O-methyl-rhamnosyl tetracyclic spinosyn pseudoaglycone | 452.925 | 1820133.15166667 | 0.00156819059290066 |
| 8.227 | NEG | 573.25415 | Lembehsterol B | 2669767.98666667 | 355301.271666667 | 0.00436796091971766 |
| 8.817 | NEG | 552.20422 | Carbazomarine A | 11339965.9283333 | 6039784.93333333 | 0.00592153287613433 |
| 6.587 | NEG | 549.30817 | 4,5-Dihydroxykleinifulgin | 50563565.17 | 20962923.71 | 0.00381327911078837 |
| 6.731 | NEG | 543.26355 | [1R-[1R*,2S*(Z),4S*,6R*,8R*(Z),9S*,10S*(Z),11R*]]-1,6-Dimethyl-9-(1-methylethenyl)-7-oxo-5,12-dioxatricyclo[9.1.0.04,6]dodecane-2,8,10-triyl ester 2-methyl-2-butenoic acid | 27308219.12 | 13388595.2783333 | 0.00223757943655369 |
| 12.555 | NEG | 541.30212 | Tricalysioside R | 49468256.9083333 | 5071715.24 | 0.000891910363773077 |
| 6.654 | NEG | 537.34314 | (+)-Milnamide A | 10380164.3083333 | 3793258.64 | 0.000487523452717921 |
| 6.622 | NEG | 535.32727 | 20,26-dihydroxyecdysone 20,22-acetonide | 25177570.0233333 | 8283066.02166667 | 0.0105227448780887 |
| 6.604 | NEG | 523.32703 | 11-Ac-(2alpha, 3beta, 5alpha, 6beta, 11alpha)-Cholest-7-ene-2, 3, 5, 6, 9, 11, 19-heptol | 9969324.30333333 | 3211105.00166667 | 0.0115811918711122 |
| 8.614 | NEG | 519.3324 | (1beta,2alpha,3beta,16beta,19beta)-1,2,3,16,19-pentahydroxyolean-12-en-28-oic acid | 36040008.9666667 | 10297020.35 | 0.00513603374036815 |
| 8.227 | NEG | 519.3324 | Verrucoside | 62066522.0316667 | 23709897.5033333 | 0.00204047260353792 |
| 6.457 | NEG | 517.28149 | Gomphoside | 21702756.0966667 | 7219301.55666667 | 0.000880973026382453 |
| 8.606 | NEG | 510.28818 | 15-O-veratroyldictysine | 8085070.55666667 | 37276513.42 | 0.00637650163942561 |
| 8.13 | NEG | 509.28833 | CHEBI:68981 | 38177735.17 | 10453963.8533333 | 0.00117045441295676 |
| 6.541 | NEG | 505.31653 | Iriomoteolide 1a | 15347836.56 | 2361517.87833333 | 0.000479030572356361 |
| 9.038 | NEG | 503.33905 | 6beta-Hydroxyasiatic acid | 57786891.1533333 | 22431768.0183333 | 0.00047353160334038 |
| 6.824 | NEG | 503.30127 | Spongianolide B | 32414732.6883333 | 7835499.86 | 0.00126672496606667 |
| 8.732 | NEG | 501.32285 | 2,3-Seco-oleana-12-en-2,3,28-trioic acid\|6a-Carboxy-1-carboxymethyl-2-(1-carboxy-1-methyl-aethyl)-1,4a,4b,9,9-pentamethyl-1,2,3,4,4a,4b,5,6,6a,7,8,9,10,10a,12,12a-hexadecahydro-chrysen | 254812567.451667 | 125211054.341667 | 0.00125431813678321 |
| 8.962 | NEG | 501.32275 | Saponaceolide B | 306153176.186667 | 116735345.338333 | 7.87617805718589e-05 |
| 9.753 | NEG | 501.32257 | CHEBI:67909 | 117804944.473333 | 27284196.4633333 | 0.00412862305267578 |
| 7.49 | NEG | 501.28564 | Kadcoccilactone B | 42351969.05 | 7874417.825 | 0.000127165327360013 |
| 8.531 | NEG | 496.27405 | 2-[[(5beta,7alpha)-7-hydroxy-3,24-dioxocholan-24-yl]amino]ethanesulfonic acid | 917750.796666667 | 22826173.7133333 | 0.00518030196616594 |
| 6.41 | NEG | 491.28574 | Bu 2867TF | 83453406.2883333 | 37903038.56 | 0.00318832447874286 |
| 7.22 | NEG | 489.32352 | Homaline | 43270505.87 | 5193093.27833333 | 0.00273048712032304 |
| 11.87 | NEG | 477.30371 | Kermadecin B | 31773933.9066667 | 8716580.47333333 | 0.00103966334650098 |
| 10.449 | NEG | 475.34253 | (2alpha,3alpha,5alpha,22R,23R)-2,3,22,23-Tetrahydroxy-25-methylergost-24(28)en-6-one | 28261729.2833333 | 56651088.7566667 | 0.0111889464329774 |
| 8.227 | NEG | 473.32739 | CHEBI:69578 | 120398988.213333 | 22523420.3216667 | 0.000694574613381254 |
| 7.431 | NEG | 473.32703 | Stoloniferone S | 31311028.4016667 | 3017830.95333333 | 0.00391909263087572 |
| 7.462 | NEG | 471.31137 | Kuguacin Q | 148490506.6 | 21902442.8533333 | 1.8819436314427e-06 |
| 9.105 | NEG | 457.33167 | Baccatin | 36956453.2283333 | 8370555.76166667 | 0.000114996536883256 |
| 7.588 | NEG | 457.29678 | CHEBI:69585 | 7459803.47166667 | 2235872.36833333 | 0.00608055887505052 |
| 7.468 | NEG | 457.22772 | 3-Sulfodeoxycholic acid | 18739051.59 | 6161157.26666667 | 0.00023144276989431 |
| 7.85 | NEG | 456.24234 | clathrimide B | 200787.6275 | 26836326.0116667 | 0.0110608467901512 |
| 10.1 | NEG | 447.34811 | Irisoquin E | 18312044.3383333 | 1443896.17583333 | 0.000165966889003451 |
| 10.88 | NEG | 445.33298 | 9-hydroxycerevisterol | 77776746.6833333 | 3643920.08833333 | 0.000268195396110268 |
| 10.004 | NEG | 441.30173 | Triptocalline A | 75074579.8566667 | 6952642.10666667 | 5.43425521143714e-05 |
| 8.687 | NEG | 429.29663 | Homaxisterol B1 | 369476917.126667 | 126780731.658333 | 0.00126171844250554 |
| 8.9 | NEG | 428.17447 | mecambridine | 18892006.0616667 | 9125059.65833333 | 0.0012050998554808 |
| 5.068 | NEG | 425.18195 | cis-1,2,3,5-trans-4,6-inositol-2,3,6-triangelate | 35394951.6966667 | 16084792.7833333 | 0.00300747897657512 |
| 7.041 | NEG | 423.20694 | Nigakilactone H | 34185088.0633333 | 7790850.17666667 | 0.00698094394234523 |
| 7.075 | NEG | 421.19061 | Phe-Trp-Ala | 16254412.1866667 | 4524394.035 | 0.00675691518000905 |
| 8.438 | NEG | 415.28113 | CHEBI:68421 | 190080900.298333 | 57802704.52 | 0.000119924133404504 |
| 8.561 | NEG | 415.24823 | salvileucolidone | 2308484.45 | 11244103.85 | 0.000707029842616072 |
| 8.356 | NEG | 407.11624 | SR-Podolactone D | 8968650.85333333 | 450909.090833333 | 0.00309534634556052 |
| 9.259 | NEG | 397.30713 | astrogorgol H | 256764.963333333 | 3986044.27666667 | 6.79051159072932e-05 |
| 5.731 | NEG | 397.22333 | Shitakigenin | 16326802.9316667 | 9818514.92833333 | 0.000290681377161444 |
| 4.966 | NEG | 397.18655 | Cinncassiol E | 33646539.2116667 | 18008117.5183333 | 0.0028340493194452 |
| 8.591 | NEG | 395.24426 | ent-13R,15-Dihydroxy-14S-acetoxy-1(10)-halimen-18-oic acid | 4871003.055 | 12846276.9316667 | 0.00662503699886983 |
| 6.554 | NEG | 393.19513 | trans-4-[4-(4-Amino-7,7-dimethyl-7H-pyrimido[4,5-b][1,4]oxazin-6-yl)phenyl]cyclohexaneacetic acid | 199196728.356667 | 100517529 | 0.00983344076692264 |
| 5.311 | NEG | 388.14325 | HMS1667D09 | 1741092.513 | 15463065.7616667 | 0.00217575331740294 |
| 5.547 | NEG | 385.09277 | 7-[3,5-dihydroxy-4-(4-hydroxyphenyl)-2-methoxyphenyl]-3-methoxy-3,4-dihydrooxepine-2,5-dione | 36765604.8883333 | 17567036.2133333 | 0.00808528772268705 |
| 7.52 | NEG | 383.2085 | 13,14-dihydro-15-oxo-20-carboxy-PGF2alpha | 6050614.175 | 3683111.71166667 | 0.00892897398287944 |
| 11.888 | NEG | 383.18927 | Bortezomib | 58819410.8216667 | 29466207.9433333 | 0.00577004375710988 |
| 5.547 | NEG | 381.19208 | 12-Oxo-20-trihydroxy-leukotriene B4 | 33463031.5866667 | 19559177.8816667 | 0.00348526878713761 |
